# Paraxanthine safety and comparison to caffeine

**DOI:** 10.3389/ftox.2023.1117729

**Published:** 2023-02-02

**Authors:** Sandra K. Szlapinski, Andrew Charrette, Najla Guthrie, Corey J. Hilmas

**Affiliations:** KGK Science Inc, Division of Client Services, Department of Regulatory Affairs, London, ON, Canada

**Keywords:** paraxanthine, 1,7-dimethylxanthine, caffeine, methylxanthine, preclinical, rodent, safety assessment, toxicology assessment

## Abstract

**Introduction:** Caffeine, one of the most ubiquitous ingredients found in beverages and other ingested food products, has a long history of safe use. As a member of the methylxanthine class of stimulants, caffeine is not devoid of unwanted side effects at any serving level. Caffeine safety has been the subject of a safety workshop by FDA and the Institute of Medicine in the past decade. Thus, investigation into an alternate stimulant with similar pharmacology but improved safety is warranted. Paraxanthine (1,7-dimethylxanthine) is the predominant metabolite of caffeine in humans with similar stimulant properties. The few toxicity studies that are available for paraxanthine suggest that the molecule is relatively safe, although thorough characterization of its safety is required prior to widespread incorporation into foods/beverages.

**Methods:** The aim of this study was to evaluate the toxicity of paraxanthine (Rarebird, Inc.) relative to caffeine through a battery of toxicological studies conducted in accordance with international guidelines. These studies evaluated the potential mutagenicity (bacterial reverse mutation, *in vitro* mammalian chromosomal aberration), genetic toxicity (*in vitro* mammalian cell gene mutation) and acute, sub-acute and sub-chronic oral toxicity of paraxanthine in Sprague Dawley rats.

**Results/Discussion:** There was no evidence of genetic toxicity or mutagenicity in the *in vitro* studies. An acute oral LD_50_ of 829.20 mg/kg body weight (bw) was established. There was no mortality or treatment-related adverse effects in the 14-day repeat dose oral toxicity study, wherein rats received low, mid, or high doses of paraxanthine (50, 100, or 150 mg/kg bw, *n* = 5 rats/sex/group). The same findings were observed in the subchronic repeat-dose 90-day oral toxicity study at daily doses of paraxanthine of 100, 150, or 185 mg/kg bw which were compared to caffeine at 150 or 185 mg/kg bw (*n* = 10 animals/sex/group). However, mortality was reported in two animals in the high dose caffeine-treated animals. Therefore, the no observed adverse effect level (NOAEL) from the 90-day study was determined to be 150 mg/kg bw for caffeine and 185 mg/kg bw for paraxanthine for both male and female Sprague Dawley rats. These findings may suggest that paraxanthine could be a safer alternative to caffeine in humans.

## 1 Introduction

Caffeine, a Central Nervous System (CNS) stimulant of the methylxanthine class, is naturally-derived from the leaves, seeds, and fruits of many plants, including tea leaves, cocoa beans, coffee beans, guarana, and kola nuts. Caffeine has been “generally recognized as safe” or GRAS since 1959 at a limit of 0.02 percent (200 ppm) in cola-type beverages (Institute of Medicine, 2014), but the serving level has increased as well as its direct addition to a wide variety of food products outside of colas, including ice cream, shots, gum, and energy bars. To this end, total daily consumption and exposure to caffeine has slowly increased globally.

Mean caffeine intake in the United States ranges between 25 mg among two- to 11-year-old children to more than 200 mg in older adults. In United States adults 50–59 years of age, the 90th percentile intake of consumption was estimated at 450 mg/day in 2014 (Institute of Medicine, 2014). Caffeine is typically consumed in the form of coffee. It is often promoted to combat fatigue, promote energy levels (Evans et al., 2022), improve mental alertness (Chen et al., 2020), and suppress appetite (Sirotkin and Kolesárová, 2021). Despite these purported benefits, caffeine intake has also been associated with adverse effects among certain consumers sensitive to the stimulant. These effects can be explained in part due to differences in caffeine metabolism, resulting in subpopulations of individuals being classified as either slow or fast caffeine metabolizers (Cornelis et al., 2016). Differentiation into these subgroups occurs based on polymorphism of the expression of CYP1A2, the predominant enzyme involved in caffeine metabolism (Chen et al., 2015). Differences in expression of CYP1A2 determine how quickly caffeine will be metabolized and eliminated from the body, ultimately influencing potential caffeine-induced side effects when caffeine is present in excess in the body. While fast metabolizers often report few side effects following caffeine use, slow caffeine metabolizers are more likely to suffer from caffeine-induced tachycardia and insomnia due to excessive caffeine intake. Similarly, certain individuals are more likely to experience caffeine-induced anxiety, which is also based on a genetic predisposition. Studies have suggested that these effects are mediated at the level of the adenosine receptor, whereby caffeine can produce anxiogenic effects by antagonizing adenosine at A1 and A2A receptors (Alsene et al., 2003; Childs et al., 2008). Evidence of single nucleotide polymorphisms (SNPs) in genes for adenosine receptors may affect these responses to caffeine, as they have been associated with greater increases in anxiety following caffeine intake. Moreover, some studies suggest more serious health risks among slow caffeine metabolizers, such as an increased risk of hypertension and acute myocardial infarction with increased coffee consumption (Cornelis et al., 2006; Palatini et al., 2009). As such, an alternative stimulant with similar benefits and less toxicity is warranted.

Paraxanthine (1,7-dimethylxanthine) is a metabolite of caffeine that can be found naturally in small amounts in some foods including green coffee beans, cotyledonary leaves of *Coffea arabica, Theobroma cacao* fruits*,* roasted coffee beans, citrus flowers and Sicilian orange flower honey (Waller et al., 1983; Kappeler and Baumann, 1985; Kretschmar and Baumann, 1999; Zheng X.-Q. et al., 2004; Arnaud, 2011; Bothiraj et al., 2020; Garg et al., 2021). However, it is predominantly found as the major metabolite of caffeine, accounting for approximately 70%–72% of ingested caffeine (Stavric, 1988). Paraxanthine has been shown to have several of the same nootropic benefits as caffeine, including improved cognition, short-term memory, sustained attention in heathy adults (Xing et al., 2021; Yoo et al., 2021) and beneficial cognitive effects in animal models of Parkinson’s disease (Xu et al., 2010; Xing et al., 2021; Yoo et al., 2021). Moreover, the beneficial impacts on physical endurance and sports performance that have been observed with caffeine use (Guest et al., 2021) have also been suggested for paraxanthine (Jäger et al., 2022). Mechanistically, some of these effects are mediated by a similar mechanism for both caffeine and paraxanthine, which act as antagonists of adenosine receptors A1 and A2a (Snyder et al., 1981; Logan and Carney, 1984; Ferré et al., 1991; Orrú et al., 2013). Despite these similarities in mechanisms, it has been reported that there are potential differences in adenosine receptor binding affinity, as paraxanthine exhibited higher binding potencies for adenosine A1 and A2A receptors in equine forebrain (Chou and Vickroy, 2003) and a stronger locomotor activating effect in rats (Orrú et al., 2013) relative to caffeine. Furthermore, paraxanthine, but not other methylxanthines (caffeine, theobromine, theophylline) has been shown to potentiate nitric oxide neurotransmission; which has been associated to increased blood flow and subsequent changes in aerobic exercise performance and cardiovascular health (Ferré et al., 2013). Since clinical studies administering caffeine and paraxanthine to healthy subjects demonstrated that caffeine produces a greater increase in diastolic blood pressure relative to paraxanthine (Benowitz et al., 1995), it is plausible that the relatively lower blood pressure increase following paraxanthine intake compared to caffeine could be due to paraxanthine’s effects on nitric oxide production. Paraxanthine is also unique from other methylxanthine stimulants in that it seems to possess anxiolytic activity (Costentin et al., 2009; Okuro et al., 2010). Although human toxicity studies on paraxanthine are scarce, the few studies that have been conducted in humans demonstrate a lack of adverse events (Xing et al., 2021; Yoo et al., 2021). In fact, an improved safety profile for paraxanthine in comparison to caffeine has been suggested based on evidence from preclinical *in vitro* and *in vivo* studies (Weinstein et al., 1975; Renner, 1982; Nakatsuka et al., 1983; York et al., 1986; Stavric, 1988; Institute of Medicine (U.S.), 2001; Gressner et al., 2009; Monteiro et al., 2016). These studies include reports that paraxanthine has low abuse liability and does not mediate the abuse-related effects of caffeine (Lazenka et al., 2015); in addition to being less clastogenic relative to caffeine (Weinstein et al., 1975), it causes less DNA damage than caffeine (Renner, 1982); exhibits less cell toxicity compared to caffeine (Gressner et al., 2009); and results in a lower incidence of malformed fetuses and resorption rates following *in utero* exposure (Nakatsuka et al., 1983; York et al., 1986). Moreover, results from a preclinical safety assessment of paraxanthine (ENFINITY™) produced by Ingenious Ingredients, L.P. demonstrated a lack of mutagenicity and genetic toxicity (Purpura et al., 2021). Furthermore, paraxanthine did not result in mortality or toxicity in 28- or 90-day repeated-dose oral toxicity studies (up to 300 mg/kg bw/day) (Purpura et al., 2021). Therefore, the no observed adverse effect level (NOAEL) was determined to be ≥ 300 mg/kg bw/d, the highest dose tested, for both male and female Wistar rats.

Given that many individuals struggle with the untoward consequences of caffeine use, paraxanthine represents a potentially attractive methylxanthine substitute for achieving similar stimulant effects with an improved safety profile over caffeine, based on the observations described above from several studies (e.g., paraxanthine has anxiolytic activity, and relative to caffeine it has low abuse liability, less clastogenicity and cytotoxicity; and lower embryo toxicity). However, in order to justify the use of paraxanthine as a food ingredient or dietary ingredient for use in dietary supplements as a caffeine alternative, its safety profile must be thoroughly characterized and compared to that of caffeine. In the United States, the addition of new food ingredients into the market requires that the ingredient be either an approved food additive or GRAS for its intended use. Alternatively, a New Dietary Ingredient (NDI) entering the marketplace must be notified to FDA prior to marketing in dietary supplements. These premarket gates ensure that the novel food ingredients and new dietary ingredients have been assessed to minimize safety risks to consumers.

While a general toxicity profile for paraxanthine has supported a lack of toxicity, it remains unclear how the potential toxicity of paraxanthine compares to the caffeine molecule. As such, the aim of this study was to independently evaluate the safety of paraxanthine (Rarebird Inc.) consumption and compare it to the safety profile of caffeine consumption using the same animal model through a battery of toxicological studies conducted in accordance with international guidelines, including mutagenicity (bacterial reverse mutation and *in vitro* mammalian chromosomal aberration), genotoxicity (*in vitro* mammalian cell gene mutation) and acute, sub-acute and sub-chronic oral toxicity studies.

## 2 Materials and methods

### 2.1 Test articles

Paraxanthine (1,7-dimethylxanthine, CAS 611-59-6) was provided by Rarebird, Inc. (San Leandro, CA) and manufactured by Shanghai Bettersyn Biotech Co., Ltd. Batch lot numbers of paraxanthine used in these studies were: 20-04A, 20-10A, and 20-12A. Certificates of analyses reported lot purities of 98.6-99.56%. The comparator compound, caffeine (1,3,7-trimethylpurine-2,6-dione, CAS 58-08-2, batch lot number 190224-P-033), was provided by Derivados Industrializados del café, S.A. (Domicilio Conocido, San Pablo Coapan Naolinco, VE 91400, Mexico). Based on results from preformulation trials performed in an analytical validation study, Hydroxypropyl methylcellulose [HPMC, 1% (w/v)] prepared in ultrapure water type-1 was used as a vehicle for preparation of the formulations of paraxanthine and caffeine. The formulations were prepared fresh prior to administration, and dose confirmation analysis was performed using HPLC to verify the concentrations. All Experiments were performed at Anthem Biosciences Pvt. Ltd, Department of Preclinical Research, Bommasandra, Bangalore.

### 2.2 *In vitro* mutagenicity and genetic toxicity studies

#### 2.2.1 Ames test

This study was conducted in accordance with OECD guidelines (No. 471, 2020) and in compliance with the OECD Principles of Good Laboratory Practice (GLP) (No. 1, 1997). Reagents and media were prepared as per the guideline requirements without any change in composition (OECD, 2020). Test strains were selected as per OECD 471 guidelines, and included *Salmonella typhimurium* TA98, TA100, TA102, TA1535, and TA1537 (Molecular Toxicology, Inc.).

The mutagenicity study was carried out at concentrations of 3,000.0, 949.5, 300.0, 94.9 and 30.0 µg of paraxanthine/plate using the pre incubation method (involving a pre incubation period with a 30-min incubation at 35 °C prior to plating) followed by the Standard Plate Incorporation method (no pre incubation period) with and without a metabolic bioactivation system (Rat liver S9 microsomal fraction). The assays were performed in triplicate, and controls were tested simultaneously (vehicle, positive and sterility controls). Bacterial revertant colonies were counted manually after a 48–72 h incubation at 37°C ± 1 °C.

To assess mutagenicity, the mutagenicity factor (number of revertants in the test item ÷ number of revertants in the vehicle) was used. For strains TA98, TA 1535 and TA 1537, at least a 3-fold increase in colonies compared to the vehicle control value was required to indicate a mutagenic effect. For strains TA 100 and TA 102, a 2-fold increase in colonies compared to the vehicle control was considered indicative of a mutagenic effect. The test item was also considered a mutagen if a reproducible, dose-related increase in the number of revertant colonies in one or more strains was observed.

#### 2.2.2 *In vitro* mammalian cell gene mutation test

This study was conducted in accordance with OECD guidelines (No. 490, 2016) (OECD, 2016) and in compliance with the OECD Principles of Good Laboratory Practice (GLP) (No. 1, 1997). Cultured mouse lymphoma cells (L5178Y TK ± 3.7.2C) at 6 × 10^6^ cells/culture (2 replicates per condition) were treated with paraxanthine (300, 150, 75 and 37.5 μg/ml), vehicle (1% DMSO), Benzo [a]pyrene (2.5 μg/ml, positive control in the presence of metabolic activation), or 4-Nitroquinoline N-Oxide (0.1 μg/ml, positive control in the absence of metabolic activation) for ∼4 h in the absence or presence of the metabolic activation system (S9). There was a 2-day expression period for the cultures, which were then plated in the absence of Trifluorothymidine (TFT) at ∼1.6 cells/well (2 plates/culture) for ‘non-selection’ and in the presence of TFT (3 μg/mL) at ∼2000 cells/well (4 plates/culture) for ‘TFT-selection’. Mutant enumeration was performed between 10 and 12 days of incubation.

#### 2.2.3 Chromosome aberration

This study was conducted in accordance with OECD guidelines (No. 475, 2014) (OECD, 2014) and in compliance with the OECD Principles of Good Laboratory Practice (GLP) (No. 1, 1997). Male (n = 35) and female (n = 35) Sprague Dawley (CD (SD) IGS) rats (6–7 weeks old at start of treatment) were sourced from Hyalasco Biotechnology (India) and randomly assigned to five groups: vehicle containing 1% HPMC (n = 10 rats/sex), positive control group receiving cyclophosphamide (40 mg/kg i. p, n = 5 rats/sex), or 25 (n = 5 rats/sex), 50 (n = 5 rats/sex), or 100 mg of paraxanthine/kg bw (n = 10 rats/sex). All groups received their respective test item formulations by oral gavage as a single dose (dose volume of 10 ml/kg b. w.). Rats were observed for clinical signs, morbidity/mortality, and body weight during the study. All rats were treated with the metaphase arresting agent, colchicine (4 mg/kg, i. p.), 3–4 h prior to sacrifice. After sacrifice by carbon dioxide (CO_2_) inhalation, bone marrow samples were collected at 12–13 h (n = 5 rats/sex from groups 1–5) or 24 h (n = 5 rats/sex from groups 1 and 5) from the last administration. Bone marrow samples were collected from tibia and femur, and processed for histological examination. The slides (4 slides/rat) were stained with giemsa solution (10% v/v) and scored for chromosomal and chromatid aberration. At least 200 readable metaphase cells were scored per rat for structural abnormality. Cytotoxicity was also evaluated by calculating the mitotic index for each animal (1,000 cells/animal).

### 2.3 *In vivo* animal studies

#### 2.3.1 Animal ethics

All studies involving animals were reviewed and approved by The Institutional Animal Ethics Committee (IAEC). Approval protocol numbers for each of the studies are as follows: IAEC protocol numbers ABD/IAEC/PR/209–20–23 (*in vivo* mammalian chromosome aberration test); ABD/IAEC/PR/202–20–23 (maximum-tolerated dose study); ABD/IAEC/PR/203–20–23 (14-day study); and ABD/IAEC/PR/167–18–21 (90-day study). The care of animals was in compliance with the regulations of the Committee for the Purpose of Control and Supervision of Experiments on Animals (CPCSEA) guidelines for laboratory animals published in the Gazette of India, 1998 and the Association for Assessment and Accreditation of Laboratory Animal Care International (AAALAC).

#### 2.3.2 Animals, housing conditions and diet

Male and/or female Sprague Dawley [CD (SD)IGS] were sourced from Hylasco Biotechnology (India) Pvt. Ltd, Telangana and acclimated prior to randomization. The health status of all rats was assessed by a veterinarian prior to study initiation to ensure only healthy animals were used. The animals were housed under standard laboratory conditions, in an environmentally monitored and air-conditioned room with adequate fresh air supply (10–15 air changes per hour). Between the three *in vivo* studies, room temperature and relative humidity was in the range of 19.2°C–24.5 °C and 41%–70%, respectively, over the course of the study period with a 12-h light and dark cycle. Animals were housed in a standard polycarbonate cage and clean autoclaved corncob was provided as bedding material. Water and gamma irradiated feed (Altromin spezialfutter Gmbh and co. KG) were provided *ad libitum*.

#### 2.3.3 Acute oral toxicity study

This study was conducted in accordance with OECD guidelines (TG 423 OECD Guideline for Testing of Chemicals. Acute Oral Toxicity, exclusions of dose levels and GHS classification) (OECD, 2002) and in compliance with the OECD Principles of Good Laboratory Practice (GLP) (No. 1, 1997). Female Sprague Dawley rats (n = 30, 8–12 weeks old at the time of treatment) were fasted overnight and treated in a stepwise procedure (n = 3 animals/group/step) by oral gavage as follows: paraxanthine at a dose of 100 mg/kg, 125 mg/kg, 200 mg/kg, 375 mg/kg, 500 mg/kg, 750 mg/kg, 1,000 mg/kg, 3,750 mg/kg, or 5,000 mg/kg (dose volume of 10 mL/kg body weight). A vehicle control group received 1% (w/v) Hydroxypropyl methylcellulose solution prepared in ultrapure water type 1. The absence or presence of compound-related mortality of the animals dosed at one step determined the dose for the next step. After administration, all animals were observed at 20–30 min, 1 h ± 10 min, 2 h ± 10 min and 4 h ± 10 min, 6 h ± 10 min post dose; once daily for clinical signs and twice daily for mortality/morbidity for 14 days. Body weights were also recorded throughout the study. On day 15, surviving animals were euthanized by CO_2_ and subjected to gross necropsy examination. All animals that were found dead or moribund were euthanized and also subjected to gross necropsy examination.

#### 2.3.4 Repeat-dose 14-day oral toxicity study

This study was conducted in compliance with the OECD Principles of Good Laboratory Practice (GLP) (No. 1, 1997). Both male and female rats (n = 20 animals/sex, 7–8 weeks of age at the time of treatment) were randomly assigned to receive vehicle control (HPMC 1% (w/v) prepared in ultrapure water type 1), or a low (50 mg/kg bw), mid (100 mg/kg bw) or high (150 mg/kg bw) dose of paraxanthine (n = 5 rats/sex/group). The test items were administered by oral gavage once daily for 14 consecutive days (at a dose volume of 5 mL/kg b. w.). All animals were observed for clinical signs once daily, mortality/morbidity twice daily and a detailed clinical examination was conducted once a week. Similarly, body weights and feed consumption were recorded at weekly intervals. At the end of the experimental period (day 15), blood samples (from the retro-orbital plexus under mild isoflurane anesthesia) were collected from overnight fasted animals for hematology, coagulation and clinical chemistry analysis. After euthanasia by CO_2_, the animals underwent gross pathology examination and specified organs were collected for histopathological evaluation using hematoxylin and eosin. Examination of the external surface of the body, all orifices, cranial, thoracic and abdominal cavities and their contents was also performed. Since treatment related lesions were not observed in the high dose group, histopathological examination was performed only on all collected tissues from the vehicle control and high dose groups.

#### 2.3.5 Repeat-dose 90-day oral toxicity study

**FIGURE 1:**
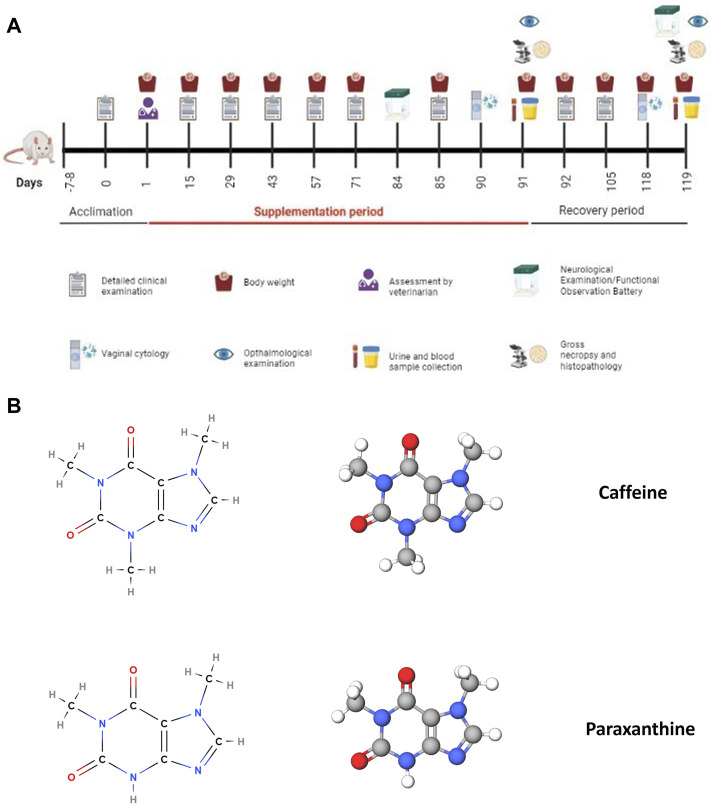
**(A)** Timeline and experimental procedures for the subchronic 90-day oral toxicity study. This figure was created using BioRender. **(B)** Chemical structures of caffeine and paraxanthine, the test items that were administered in the oral toxicity study to compare their safety profile. These figures were created using MolView.

This study was conducted in accordance with OECD guidelines (OECD test guideline 408 (adopted 25 June 2018) (OECD, 2018) and in compliance with the OECD Principles of Good Laboratory Practice (GLP) (No. 1, 1997). One-hundred and fifty male and female (75 males and 75 females) Sprague Dawley rats (5–6 weeks of age at the time of treatment) were randomly assigned to receive vehicle (HPMC 1% (w/v) prepared in ultrapure water type 1), paraxanthine (100, 150, or 185 mg/kg bw), or caffeine (150 or 185 mg/kg bw, n = 10 animals/sex/group) daily for 90 days by oral gavage (dose volume of 5.0 mL/kg body weight). An additional group of high dose paraxanthine (185 mg/kg bw), high dose caffeine (185 mg/kg bw animals) and vehicle treated animals (*n* = 5 animals/sex/group) served as a recovery group wherein the test items were withdrawn from the animals for 28 days. All animals in the study were observed for signs of toxicity until the end of the study period, including mortality/morbidity, clinical signs, body weight and feed consumption. Ophthalmological examinations (weeks 13 and 17), cage rotation and neurological examination/functional observation battery tests (weeks 12 and 17) were also conducted. The functional observation battery tests included: Home cage measurements, Handheld measurements, Open field measurements, Reflex measurements, Neuromuscular measurements and Physiological measurements. Clinical pathology measurements included Urinalysis (week 13 for the main study group, week 17 for recovery groups), Hematology, Clinical Chemistry, Coagulation and Hormonal Analysis from treated animals on day 91 or recovery group animals on day 119 were assessed. Terminal vaginal cytology was also evaluated on days 90 and 118. Serum T4, T3 and TSH were quantified *via* ELISA kits with standard samples run in duplicate (Endocrinetech, 021,921). All surviving animals were euthanized by CO_2_ on days 91 and 119 for main study and recovery groups, respectively. Detailed gross necropsy included the examination of the external surface of the body, all orifices, cranial, thoracic and abdominal cavities and their contents. Upon euthanasia, several organs from the main study groups (vehicle and high dose paraxanthine and caffeine-treated animals) were weighed and processed for histopathological examination with haematoxylin and eosin. A timeline of the experimental procedure is demonstrated in Figure 1.

### 2.4 Statistics

Statistical analyses were performed using Graph Pad Prism version 5.03, Graph Pad Software. Parameters were analyzed by either an unpaired Student’s t-test (two-tailed), or a one-way ANOVA followed by Dunnett’s *post hoc* test depending on the experimental groups being compared. Fisher’s exact test was used to analyze total aberrant cells per group compared to the vehicle control in the chromosome aberration assay. LD_50_ was estimated using Probit Analysis in Microsoft Excel. Male and female data were considered separately for analysis. Outliers were calculated using Grubbs test. All analysis and comparisons were evaluated at a 95% level of confidence (*p* < 0.05).

## 3 Results

### 3.1 *In vitro* studies

#### 3.1.1 Ames test

**FIGURE 2:**
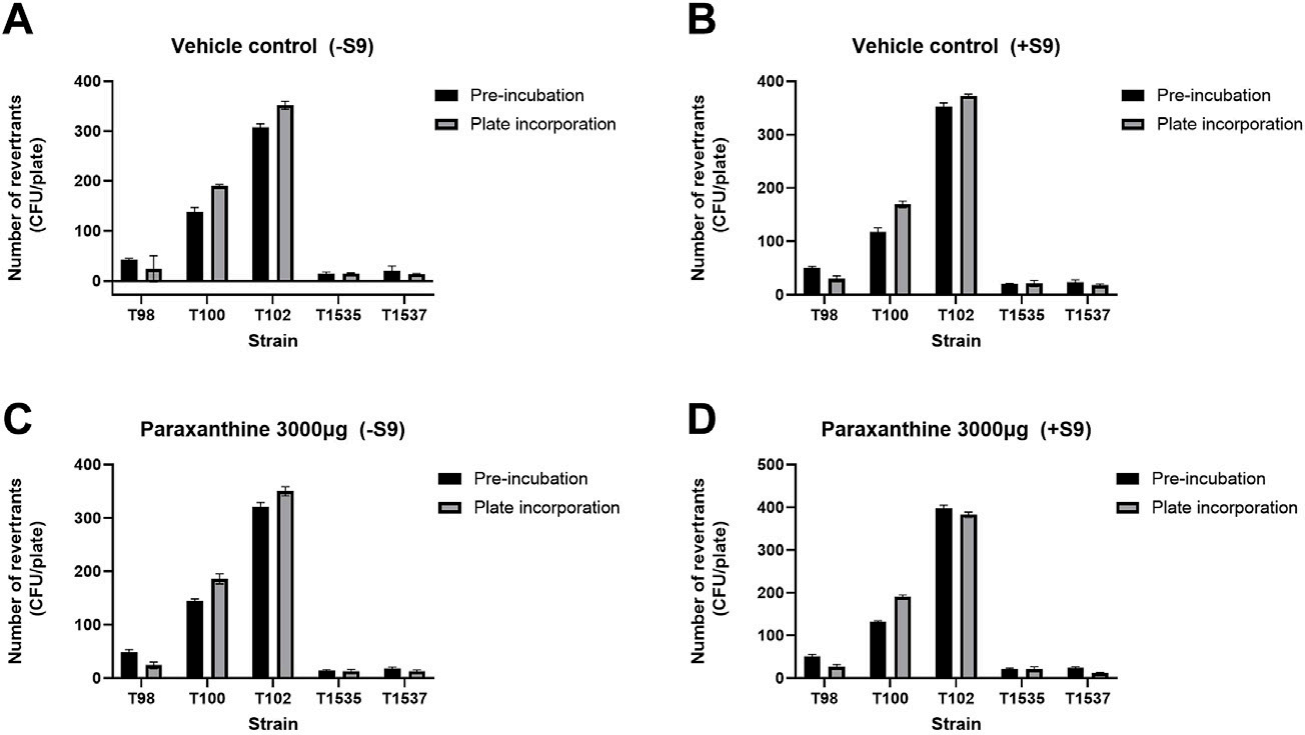
Number of His + revertrants in the pre-incubation and plate incorporation method with all strains of *Salmonella typhimurium* for the vehicle control in the **(A)** absence and **(B)** presence of rat liver S9 metabolic activation; and with the highest concentration of paraxanthine (3,000 µg/plate) in the **(C)** absence and **(D)** presence of rat liver S9 metabolic activation.

The potential mutagenicity of paraxanthine was investigated by testing its’ ability to revert strains of *Salmonella typhimurium* in the absence and presence of metabolic bioactivation (S9). A preliminary toxicity study demonstrated that the highest concentration of paraxanthine (3,000.0 µg/plate) was not cytotoxic in either the plate incorporation method or pre incubation method with/without S9. In the main experiment, the mean number of His + revertants in paraxanthine-treated cells was comparable to that of the vehicle (DMSO) controls in the plate incorporation method and pre incubation method with/without S9 (Figures 2A–D). On the other hand, the positive controls showed mutagenic responses with and without S9 activation, as there was a significant increase in the number of His + revertants with all strains in the plate incorporation method and pre incubation method, with the mutagenicity factor ranging between 2.3 and 56.1. Therefore, paraxanthine was non-mutagenic at the tested concentrations (3,000.0, 949.5, 300.0, 94.9 and 30.0 µg/plate).

#### 3.1.2 *In vitro* mammalian cell gene mutation test

The potential mutagenic activity of paraxanthine was also investigated using L5178Y TK ± Mouse Lymphoma cells, by assessing the capability of paraxanthine to induce forward gene mutations and/or structural chromosomal damage at the TK locus (TK^+/−^ → TK^−/−^). Cytotoxicity was assessed in a preliminary experiment, wherein no dose-related decrease was observed in the relative total growth at any of the tested concentrations of test item in absence (∼4 h) and presence (∼4 h) of metabolic activation system. Based on these results, concentrations of 300, 150, 75 and 37.5 μg/mL were selected for the main mutagenicity study.

**FIGURE 3:**
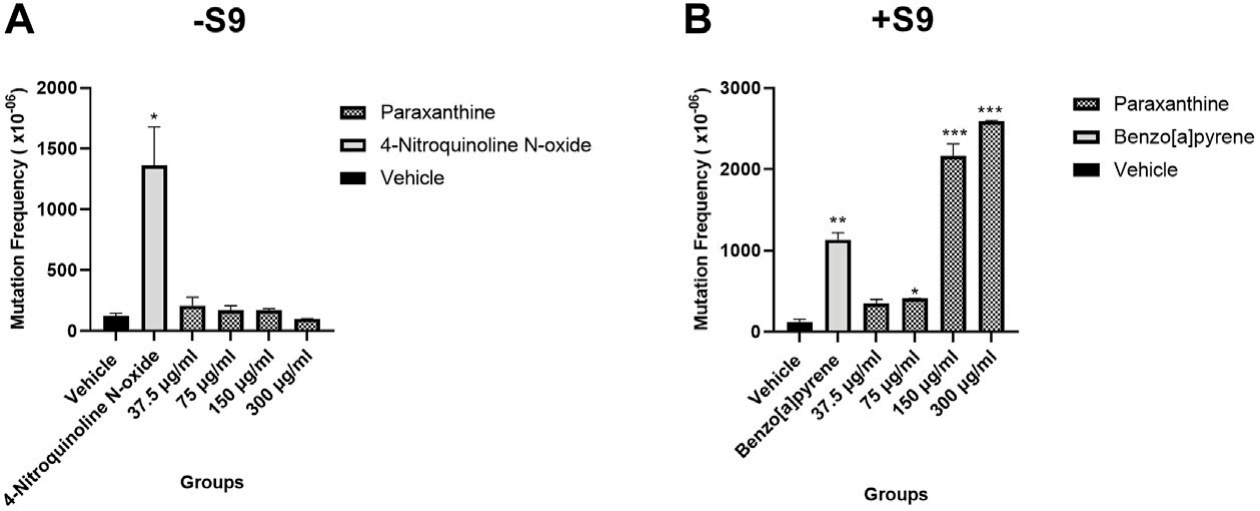
Mutant frequency in the **(A)** absence and **(B)** presence of S9. Statistically significant from the vehicle control at *p* < 0.05 (*), *p* < 0.01 (**), *p* < 0.001 (***).

In the absence of S9, there were no significant differences in the frequency of mutants observed at any of the tested concentrations of paraxanthine (Figure 3A). On the other hand, in the presence of S9, there was a statistically significant increase in the frequency of TK mutants at 75, 150 and 300 µg of paraxanthine/mL (Figure 3B). There was also a dose dependent increase of TK gene mutation across the tested doses in the presence of S9 (*p* < 0.05). The results demonstrate that at concentrations up to 300 μg/mL, paraxanthine induces TK gene mutation and/or chromosomal mutations at the TK locus of cultured mouse lymphoma cells in the presence of metabolic activation.

#### 3.1.3 *In vivo* mammalian bone marrow chromosomal aberration test

This study was conducted to evaluate the chromosomal aberration potential of paraxanthine in the bone marrow of rats treated by oral gavage. All doses of paraxanthine were well-tolerated, as there was no mortality/morbidity, no changes in body weight or body weight gain, and no adverse clinical signs of toxicity observed. While the positive control group showed a significant increase in the number of aberrations per cell and total number of cells with aberrations compared to vehicle control rats, in addition to a lower mitotic index compared to the vehicle control rats, there were no significant differences between the groups treated with varying doses of paraxanthine compared to the vehicle control group (Table 1). Therefore, the results indicate that paraxanthine does not result in chromosomal aberration or toxicity to the bone marrow of Sprague Dawley rats at doses up to 100 mg/kg bw.

**TABLE 1:** Summary results of the bone marrow chromosome aberration test. Statistically significant compared to the control group at p < 0.05 (*).

Group & dose (mg/kg b.w.)	% Mitotic index (mean ± SD)		Total aberrant cells (mean ± SD)		Number of aberrations per cell	
	First sampling					
	Male	Female	Male	Female	Male	Female
**Vehicle control**	12.10 ± 0.91	10.42 ± 0.41	0.00 ± 0.00	0.00 ± 0.00	0.00 ± 0.00	0.00 ± 0.00
**Cyclophosphamide (40)**	8.62* ±0.48	8.24* ±0.29	14.40* ±5.59	15.60* ±3.21	0.11* ±0.03	0.12* ±0.04
**Paraxanthine (25)**	11.84 ± 0.51	10.98 ± 0.75	0.40 ± 0.55	0.00 ± 0.00	0.00 ± 0.00	0.00 ± 0.00
**Paraxanthine (50)**	12.30 ± 0.54	11.3 ± 0.54	0.00 ± 0.00	0.00 ± 0.00	0.00 ± 0.00	0.00 ± 0.00
**Paraxanthine (100)**	11.02 ± 1.43	10.76 ± 0.77	0.00 ± 0.00	0.00 ± 0.00	0.00 ± 0.00	0.00 ± 0.00
**Second sampling**						
**Vehicle control**	10.76 ± 1.70	11.14 ± 0.59	0.20 ± 0.45	0.00 ± 0.00	0.00 ± 0.00	0.00 ± 0.00
**Paraxanthine (100)**	10.32 ± 1.54	11.12 ± 0.13	0.40 ± 0.55	0.00 ± 0.00	0.00 ± 0.00	0.00 ± 0.00

### 3.2 *In vivo* studies

#### 3.2.1 Acute toxicity: Maximum tolerated dose study

A maximum tolerated dose study was conducted to determine the acute systemic toxicity potential of paraxanthine following a single dose. There were no adverse effects on body weight or body weight gain in the animals that survived until day 15 at doses of paraxanthine up to 750 mg/kg b. w. Clinical signs of toxicity were observed at varying doses, and death was reported at 5,000, 3,750, 1,000, 750 and 500 mg/kg bw (Supplementary Table S1). Gross necropsy results demonstrated a reduced thymus size in paraxanthine-treated rats (200, 375, 500 and at 750 mg of paraxanthine/kg bw). However, these effects were postulated to be due to stress. Based on these results, the calculated single dose LD_50_ of Paraxanthine is 829.20 mg/kg b. w. under the conditions of this study.

#### 3.2.2 Repeated dose toxicity: 14-day study

**FIGURE 4:**
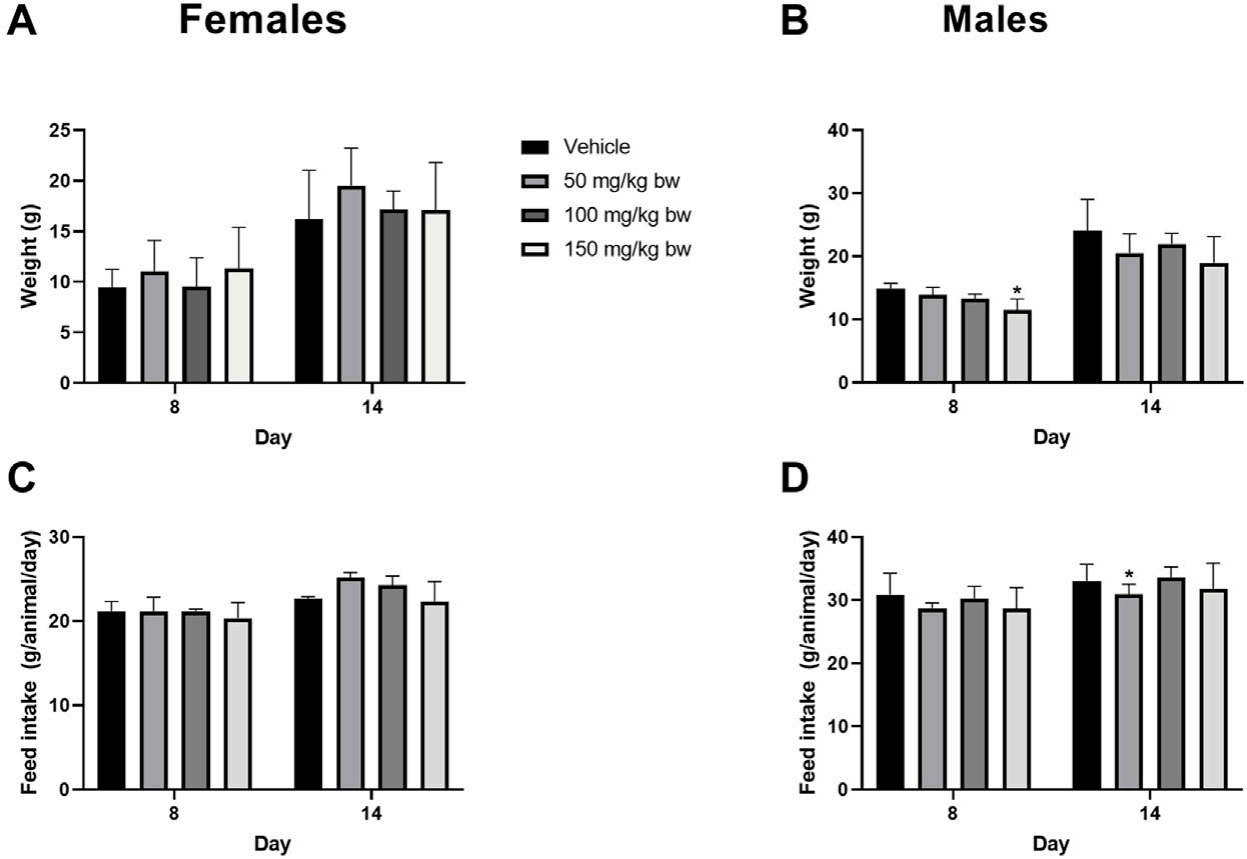
Body weight gain (%) relative to day 1 and feed consumption for females **(A ,C)** and males **(B,D)** in the repeat-dose 14-day toxicity study. Statistically significant compared to the control group at p < 0.05 (*).

Next, a 14-day repeat dose study was conducted to determine the systemic toxicity potential of paraxanthine upon repeated, once-daily administration for 14 consecutive days. Rats of both sexes showed no adverse clinical signs of toxicity, mortality, or morbidity throughout the experimental period. There were no significant differences in body weight gain in females (Figure 4A); although, body weight gain was significantly reduced (day 1–8) in the high dose males (Figure 4B). However, this effect was only observed in one sex; not observed at the second week observation; and there were no adverse changes in feed consumption (Figures 4C, D). The changes were also found to be marginal (3.42%). Therefore, the observed changes were considered as incidental and non-adverse. Similarly, a significant decrease in feed consumption at week 2 (day 8–14) in low dose males (Figure 4D) was observed and also considered to be incidental and non-adverse as the differences were observed only in one sex; no significant change was observed in the first week of observation; there were no adverse changes in body weight; and the changes were marginal.

In terms of clinical chemistry, there were significant differences in calcium, cholesterol, and ALT in the paraxanthine-treated animals (Supplementary Table S2). However, the changes were considered as non-adverse, since the relationship was non-dose dependent; the changes were observed in a single sex and were marginal compared to the control; and no changes in other correlated parameters, such as phosphorus or potassium were observed. The values were also within the normal physiological range. Similarly, toxicologically non-significant findings for hematocrit were observed in high dose paraxanthine males. The remaining clinical chemistry and hematology parameters tested were not significantly different between groups.

Upon external and internal gross pathology assessment, minimal tubular dilation in the kidney was observed in a high dose paraxanthine group male (1/5). However, the changes were considered incidental as similar lesions were observed in one vehicle male (1/5) and female (1/5). There were no significant differences in absolute or relative organ weights, or histopathological findings between the paraxanthine and vehicle treated-groups.

Clinical signs of hyperactivity and hyperreflexia were observed during post dose observations in all animals that received paraxanthine. However, all the clinical signs were found to be reversed at the next observation (approximately 3–5 h) on the same day. Since hyperreflexia is a typical response of stimulant compounds, it was not considered an adverse event.

#### 3.2.3 Subchronic repeated dose toxicity: 90-day study

A repeated dose 90-day toxicity study was performed to compare the safety profile of paraxanthine to caffeine when administered by oral gavage daily over 90 consecutive days. A recovery group was included for both test articles to investigate whether effects were persistent or reversible.

##### 3.2.3.1 Mortality/morbidity, clinical signs, vaginal cytology and ophthalmological examination

Mortality/morbidity was not observed in vehicle control and paraxanthine test item treatment groups. However, mortality was reported on days 84 and 85 in two animals in the high dose caffeine-treated group.

Paraxanthine-treated animals showed clinical signs of hyperactivity and hyper-reflexia at all doses in both sexes during the treatment period and in the high dose recovery group during the treatment period, which was expected due to the pharmacological class effect of the test item. The clinical signs were completely reversed at the next-day observation in the treatment period, and all animals displayed normal behavior during the recovery period. Similar transient abnormalities were reported in both doses of the caffeine group, wherein the animals showed hyperactivity, increased burrowing behavior and partial eyelid ptosis.

There were no treatment-related ophthalmological abnormalities in the high dose paraxanthine- or caffeine-treated groups compared to the vehicle control group upon ophthalmological examination performed at weeks 13 and 17. Since treatment-related abnormalities were not observed in the high dose group, the examination of lower dose groups was not performed. Similarly, stages of the estrus cycle for all females on days 90 and 118 demonstrated no treatment-related adverse effects as there was an even distribution of stages of the estrus cycle among all the groups.

##### 3.2.3.2 Body weight and feed consumption

**FIGURE 5:**
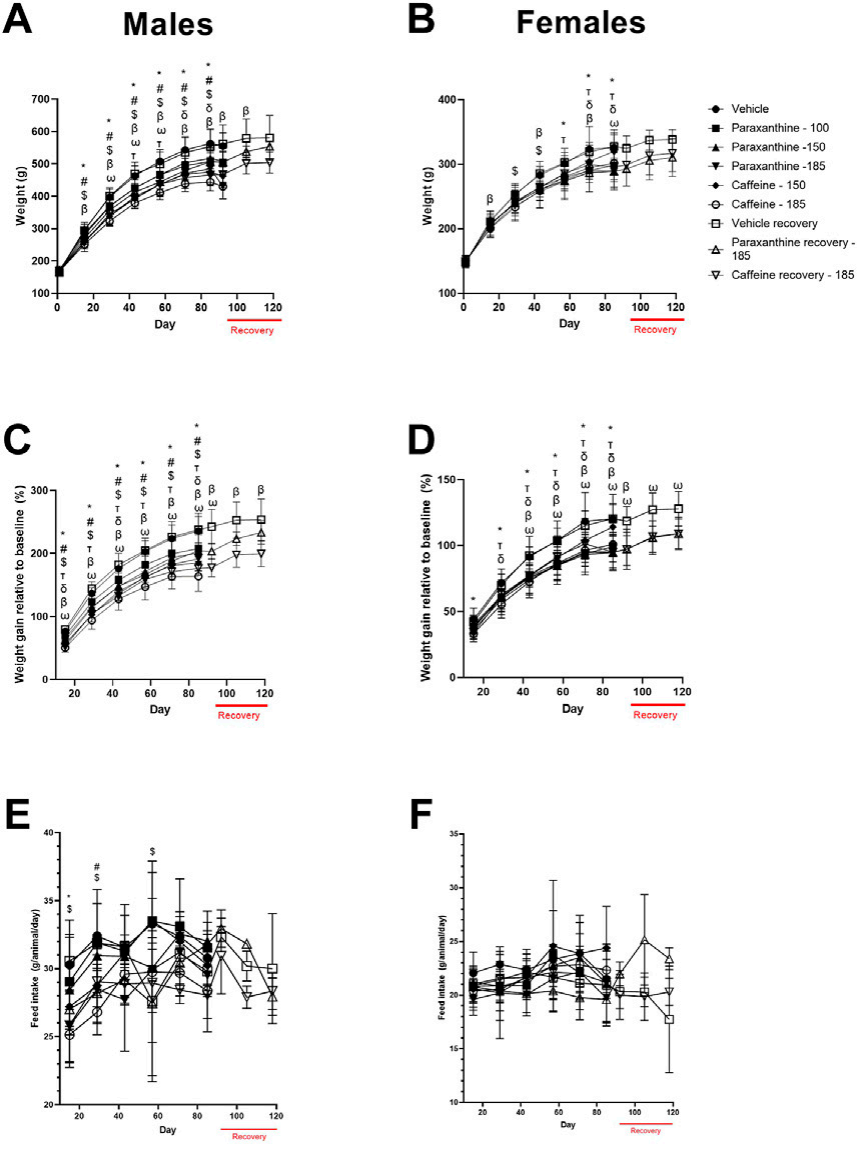
Summary of body weight **(A, B)**, body weight gain **(C, D)**, and feed consumption data **(E, F)** for males and females in the repeat-dose 90-day toxicity study. *p* < 0.05 relative to the vehicle control for 100 mg of paraxanthine/kg bw (δ); 150 mg of paraxanthine/kg bw (T); 185 mg of paraxanthine/kg bw (*); 150 mg of caffeine/kg bw (#); 185 mg of caffeine/kg bw ($); and recovery animals at 185 mg of paraxanthine/kg bw (ω) and recovery animals at 185 mg of caffeine/kg bw (β).

Body weight (Figures 5A,B) and body weight gain (Figures 5C,D) were significantly reduced at various timepoints throughout the study in both sexes of the caffeine and paraxanthine-treated rats compared to the vehicle control group. Recovery of body weights was observed in both sexes treated with both test compounds compared to the concurrent vehicle control group.

There was a significant reduction in feed consumption in high-dose paraxanthine-treated males at both doses of caffeine (Figures 5E,F). However, there were no treatment-related adverse effects on average feed consumption in females treated with caffeine or paraxanthine compared to vehicle during the experimental period.

##### 3.2.3.3 Neurological examination/functional observation battery

There was a statistically significant decrease in rearing count, urination, and foot splay in caffeine-treated groups (low and high doses), and a significant increase in defecation in the caffeine-treated groups (high dose) compared to the vehicle group (Table 2; Table 3). In the high dose paraxanthine-treated animals, there was a statistically significant decrease in urination and rearing count of the recovery group female animals (Supplementary Table S3). However, since the effects were only observed in a single sex and other parameters evaluated were found to be normal; the effects were considered as incidental.

**TABLE 2:** Summary of functional observation battery test measurements in males in the repeat-dose 90-day toxicity study. The numbers represent the number of animals showing the respective observations. *Statistically significant (p < 0.05) with respect to the control group (vehicle).

Group no.		Paraxanthine				Caffeine	
**Dose (mg/kg b.w.)**		**0**	**100**	**150**	**185**	**150**	**185**
**Number of animals**		**10**	**10**	**10**	**10**	**10**	**10**
**Open field measurement**							
**A. Gait**							
1	Normal	10	10	10	10	10	9
2	Ataxia, uncoordinated movement, excessive sway, rock, or lurch (stop and sudden movement)	0	0	0	0	0	1
3	Body drags or is flattened (animal’s ventral surface makes contact with the cart surface)	0	0	0	0	0	0
4	Limbs splayed or dragging; unable to support weight (specify hind or forelimbs)	0	0	0	0	0	0
**B. Mobility**							
1	Normal (animal moves easily around open field)	10	8	9	10	9	9
2	Increased movement (animal moves mostly continuously, rarely stopping to sniff or groom)	0	2	1	0	1	1
3	Decreased movement (reduced movement around field; movements may be sluggish)	0	0	0	0	0	0
4	None (animal does not move around field even after gently prodding)	0	0	0	0	0	0
**C. Arousal**							
1	Normal and alert with exploratory movements	10	10	10	10	8	9
2	Low; slight stupor, some head or body movements	0	0	0	0	1	0
3	Very low; stupor, little or no responsiveness to the environment	0	0	0	0	0	0
4	High; slight excitement, tense, sudden darting (sudden rapid movement) or freezing	0	0	0	0	1	1
5	Very high; hyper alert, sudden boost of running or movement	0	0	0	0	0	0
**D. Stereotype**							
1	None	10	10	10	10	7	9
2	Excessive grooming	0	0	0	0	3	0
3	Repetitive circling	0	0	0	0	0	1
4	Any other	0	0	0	0	1	0
**E. Piloerection**							
1	Absent	10	10	10	9	10	10
2	Present	0	0	0	1	0	0
**F. Clonic and tonic movement**							
1	Absent	10	10	10	10	10	9
2	Present	0	0	0	0	0	1
**G. Number of Urinations**							
**Mean**		2.50	1.50	2.90	2.50	1.50	1.40
**STDEV (±)**		3.72	1.90	2.02	2.46	1.78	1.26
**H. Number of Defecations**							
**Mean**		0.40	0.10	0.90	0.70	0.40	1.90*
**STDEV (±)**		1.26	0.32	1.90	1.25	0.70	1.40
**I. Number of rearing**							
**Mean**		10.20	9.00	10.80	7.50	5.20	6.10*
**STDEV (±)**		3.26	3.62	4.49	3.66	3.16	3.81
**J. Foot splay (cms)**							
**Mean**		7.61	8.75	7.28	6.83	8.11	6.14*
**SD (±)**		1.83	1.27	1.31	0.95	1.72	1.18

**TABLE 3:** Summary of functional observation battery test measurements in females in the repeat-dose 90-day toxicity study. The numbers represent the number of animals showing the respective observations. *Statistically significant (*p* < 0.05) with respect to the control group (vehicle).

Group no.		Paraxanthine				Caffeine	
**Dose (mg/kg b.w.)**		**0**	**100**	**150**	**185**	**150**	**185**
**Number of animals**		**10**	**10**	**10**	**10**	**10**	**10**
**Open field measurement**							
**A. Gait**							
1	Normal	10	10	10	9	10	9
2	Ataxia, uncoordinated movement, excessive sway, rock, or lurch (stop and sudden movement)	0	0	0	1	0	0
3	Body drags or is flattened (animal’s ventral surface makes contact with the cart surface)	0	0	0	0	0	1
4	Limbs splayed or dragging; unable to support weight (specify hind or forelimbs)	0	0	0	0	0	0
**B. Mobility**							
1	Normal (animal moves easily around open field)	10	6	10	7	10	9
2	Increased movement (animal moves mostly continuously, rarely stopping to sniff or groom)	0	4	0	1	0	0
3	Decreased movement (reduced movement around field; movements may be sluggish)	0	0	0	2	0	1
4	None (animal does not move around field even after gently prodding)	0	0	0	0	0	0
**C. Arousal**							
1	Normal and alert with exploratory movements	10	10	10	3	5	3
2	Low; slight stupor, some head or body movements	0	0	0	0	1	1
3	Very low; stupor, little or no responsiveness to the environment	0	0	0	2	0	0
4	High; slight excitement, tense, sudden darting (sudden rapid movement) or freezing	0	0	0	5	4	6
5	Very high; hyper alert, sudden boost of running or movement	0	0	0	0	0	0
**D. Stereotype**							
1	None	10	10	10	10	6	10
2	Excessive grooming	0	0	0	0	3	0
3	Repetitive circling	0	0	0	0	0	0
4	Any other (Dragging lower jaw against the floor)	0	0	0	0	1	0
**E. Piloerection**							
1	Absent	10	10	10	10	10	10
2	Present	0	0	0	0	0	0
**F. Clonic and tonic movement**							
1	Absent	10	10	10	10	10	10
2	Present	0	0	0	0	0	0
**G. Number of Urinations**							
**Mean**		2.70	1.20	1.50	1.20	0.70*	0.90
**STDEV (±)**		2.11	1.23	1.90	1.93	1.06	0.91
**H. Number of Defecations**							
**Mean**		0.00	0.10	0.20	0.20	0.20	0.30
**STDEV (±)**		0.00	0.32	0.42	0.42	0.42	0.67
**I. Number of rearing**							
**Mean**		11.40	14.00	13.40	7.80	9.70	9.20
**STDEV (±)**		4.60	4.14	3.81	5.55	3.40	2.90
**J. Foot splay (cms)**							
**Mean**		5.96	6.76	6.55	5.30	7.03	5.74
**SD (±)**		1.96	1.18	1.37	1.57	1.53	1.06

Based on these findings, neurological examination was extended to lower dose groups of both the test items in the daily assessment of clinical signs. Isolated cases of clinical signs such as alertness, ataxia, increased locomotor activity, excitement, and repetitive circling were observed in groups treated with the two test items; these observations were more prominent in caffeine-treated groups.

##### 3.2.3.4 Clinical pathology

Certain parameters evaluated in the clinical chemistry and hematology assessments (Table 4; Table 5) were significantly different in paraxanthine and caffeine-treated animals compared to controls. However, these changes were considered to be incidental as they were either non-dose dependent; the values remained within a normal healthy range; or differences in values occurred in only one sex. The remaining clinical chemistry parameters that were tested were not significantly different between groups (Supplementary Tables S4, S5). Similarly, there were no significant differences in hematology parameters between groups. Significant increases in urine volume of mid and high dose paraxanthine males; specific gravity of low and mid dose paraxanthine and high dose caffeine females; and urine pH of high dose caffeine females compared to controls were also observed (Supplementary Table S6). Urine volume was significantly increased in high dose recovery paraxanthine males compared to the recovery control group (Supplementary Table S7). There were no treatment-related adverse effects on clinical chemistry parameters in the recovery group animals compared to the respective vehicle control groups (Supplementary Tables S8, S9).

**TABLE 4:** Summary of clinical chemistry, hematology and hormone parameters assessed in main group males in the repeat-dose 90-day toxicity study. *Statistically significant (p < 0.05) with respect to the vehicle group.

Dose (mg/kg b.w.)	Vehicle 0		Paraxanthine - 100		Paraxanthine - 150		Paraxanthine - 185		Caffeine - 150		Caffeine - 185	
	Mean	SD	Mean	SD	Mean	SD	Mean	SD	Mean	SD	Mean	SD
**ALT (U/L)**	46.5	8.34	68.70*	17.99	58.2	12.15	64.10*	12.63	65.80*	10.5	69.56*	12.76
**AST (U/L)**	75.7	11.05	182.20*	80.74	124.60*	33.94	92.00	7.51	118.10*	13.77	113.11*	16.77
**Ca (mg/dL)**	9.69	0.23	8.68*	0.53	9.50	0.44	9.72	0.28	9.33	0.66	9.43	0.54
**Cholesterol (mg/dL)**	56.6	10.63	58.20	10.43	57.9	9.28	60.3	8.92	73.20*	17.83	74.11*	13.19
**Creatinine (mg/dL)**	0.24	0.05	0.22	0.07	0.12*	0.04	0.16*	0.07	0.23	0.05	0.34*	0.07
**GGT (U/L)**	5.30	1.06	4.80	1.32	4.10*	0.57	4.30	0.82	4.20	1.48	5.11	1.05
**Phosphorus (mg/dL)**	5.39	0.28	5.90*	0.5	6.27*	0.44	6.33*	0.36	6.51*	0.52	6.11*	0.85
**Triglycerides (mg/dL)**	55.70	23.2	46.00	16.46	28.10*	9.64	32.10*	8.06	28.90*	10.47	24.22*	6.04
**HDL (mg/dL)**	52.2	9.9	54.00	7.16	53.00	7.64	57.60	7.18	67.90*	13.67	70.56*	11.65
**LDL (mg/dL)**	8.50	1.18	10.20	1.93	12.50*	3.34	10.50	1.84	12.90*	2.92	13.44*	1.88
**Na+ (mmol/L)**	136.45	1.86	136.43	1.83	136.09	1.4	136.22	1	134.60*	1.36	135.1	0.82
**K+ (mmol/L)**	4.4	0.14	4.38	0.35	4.73	0.42	4.6	0.41	4.58*	0.2	4.31	0.15
**Cl- (mmol/L)**	104.56	1.21	104.76	1.19	104.95	0.5	105.28	0.89	103.12*	0.87	102.29*	1.66
**T3**	5.3	1.3	6.6	1.8	6.9	1.8	9.8*	2.7	7.5*	1.5	8.7*	1.1
**T4**	230.8	47.2	204.5	36.9	177.8*	36.2	173.9*	32.1	188.2*	21.4	181.7*	13.9
**WBC (10** ^**3**^ **cells/µL)**	9.32	2.13	9.85	2.53	10.86	1.99	9.98	1.78	10.45	2.01	11.65*	1.46
**RBC (10** ^**6**^ **cells/µL)**	9.56	0.35	9.22	0.71	9.48	0.36	9.58	0.39	9.08*	0.3	9.29	0.55
**MCV (fL)**	56.38	1.23	57.25	1.7	58.38*	1.43	58.35*	1.43	58.39	2.74	57.89	1.66
**MCH (pg)**	16.72	0.38	16.86	0.95	16.96	0.81	17.47	0.41	17.43*	0.86	17.34	0.41

**TABLE 5:** Summary of clinical chemistry, hematology and hormone parameters assessed in main group females in the repeat-dose 90-day toxicity study. *Statistically significant (p < 0.05) with respect to the vehicle group.

Dose (mg/kg b.w.)	Vehicle 0		Paraxanthine - 100		Paraxanthine - 150		Paraxanthine - 185		Caffeine - 150		Caffeine - 185		
	Mean	SD	Mean	SD	Mean	SD	Mean	SD	Mean	SD	Mean	SD	
**Total protein (g/dL)**	7.31	0.51	8.22*	1.43	7.12	0.43	7.03	0.53	7.31	0.26	7.19	0.56	
**Glucose (mg/dL)**	129.6	15.45	163.70*	31.42	148.1	25.07	143.9	18.16	141.6	14.14	136.1	29.5	
**Blood urea nitrogen (mg/dL)**	18.00	3.13	14.70*	3.37	14.50*	1.72	15.00	3.02	16.6	3.06	20.20	6.44	
**Cholesterol (mg/dL)**	73.5	10.31	100.20*	22.52	79.7	15.75	79.00	17.27	90.00*	14.54	94.20*	11.32	
**HDL (mg/dL)**	61.9	7.05	88.20*	19.19	73.7	12.62	73.2	14.12	83.50*	12.89	86.90*	10.42	
**LDL (mg/dL)**	6.6	0.7	11.60*	3.37	8.5	2.27	8.1	1.91	10.70*	2.41	12.80*	1.75	
**Triglycerides (mg/dL)**	75.1	53.34	37.60*	12.4	36.80*	11.71	42.30*	14.61	44.4	22.17	39.4	8.76	
**Ca (mg/dL)**	10.1	0.29	8.66*	0.71	8.91	0.83	9.58	0.33	8.63*	0.36	8.38*	0.78	
**Phosphorus (mg/dL)**	5.35	0.78	5.66	0.53	5.66	0.38	5.66	0.57	5.86	0.47	6.10*	0.61	
**GGT (U/L)**	4.5	0.53	5.00	0.82	5.4	1.17	5.3	1.16	5.80*	1.03	5.60*	1.17	
**Globulin (calc, g/dL)**	5.83	0.37	6.63*	1.07	5.77	0.49	5.66	0.43	5.89	0.23	5.83	0.45	
**T3**	4.7	0.8	6.1	1.4	6.1	1.1	8.6*	1.7	5.9*	1.0	5.3	1	
**T4**	169.6	31.6	176.7	28	178.8	24	175.9	15.8	122.8*	25.3	124.2*	48.9	
**TSH**	1.7	0.6	3.6	3.4	2.7	1.7	2.2	1	2.1	0.4	5.1*	5.1	
**Neutrophils (10** ^**3**^ **cells/µL)**	1.04	0.28	1.34	0.63	2.22	4.15	1.14	0.76	1.63*	0.57	2.09	0.9	

Furthermore, several of these findings represented non-adverse changes because they were accompanied by no histopathological changes or changes in organ weights (Tables 6; Tables 7; Tables 8). Although there were statistically significant differences in absolute organ weights of adrenals in paraxanthine treated females (mid dose); there were no changes in respective relative organ weights nor were there any associated microscopic findings. Therefore, the decrease in absolute organ weight was not considered test-item-related and the changes were determined to be incidental.

**TABLE 6:** Summary of absolute organ weights for males in the repeat-dose 90-day toxicity study. Values represent mean ± SD. *Statistically significant (*p* < 0.05) with respect to the vehicle group.

Dose (mg/kg b.w.)	Vehicle 0	Paraxanthine - 100	Paraxanthine - 150	Paraxanthine - 185	Caffeine - 150	Caffeine - 185
**Adrenals**	0.0717 ± 0.01567	0.07626 ± 0.00976	0.08255 ± 0.01855	0.07582 ± 0.01135	0.07535 ± 0.01470	0.07238 ± 0.00883
**Thymus**	0.32622 ± 0.07970	0.32662 ± 0.06751	0.32847 ± 0.05262	0.32265 ± 0.07622	0.25600 ± 0.06019	0.25013 ± 0.10435
**Spleen**	0.84338 ± 0.14572	0.88244 ± 0.13091	0.78628 ± 0.11092	0.71972 ± 0.07872	0.72820 ± 0.11576	0.65774 ± 0.12626*
**Heart**	1.63638 ± 0.15170	1.75357 ± 0.20822	1.72499 ± 0.16486	1.56995 ± 0.17801	1.51622 ± 0.15801	1.43477 ± 0.13813*
**Brain**	2.21992 ± 0.10958	2.22757 ± 0.08154	2.23672 ± 0.14605	2.18207 ± 0.11585	2.26520 ± 0.50627	2.04263 ± 0.16689
**Kidneys**	3.83966 ± 0.49104	4.05605 ± 0.53999	3.87567 ± 0.44564	3.73106 ± 0.44318	3.60848 ± 0.52868	3.24983 ± 0.34649 *
**Liver**	17.07472 ± 2.49331	15.68385 ± 1.60798	15.40151 ± 1.21252	14.95921 ± 1.88977	15.49629 ± 1.45088	13.56774 ± 0.95395 *
**Testes**	3.53203 ± 0.41386	3.52392 ± 0.31373	3.66054 ± 0.23105	3.46814 ± 0.28071	3.28702 ± 0.16618	3.07738 ± 0.25287*
**Epididymides**	1.57323 ± 0.16566	1.58983 ± 0.22349	1.59500 ± 0.12212	1.41477 ± 0.09983	1.47099 ± 0.15599	1.29733 ± 0.17271*
**Thyroid and parathyroid**	0.03479 ± 0.00504	0.03301 ± 0.00564	0.03455 ± 0.00684	0.03753 ± 0.00941	0.03212 ± 0.00418	0.03873 ± 0.00488*
**Pituitary**	0.02065 ± 0.00491	0.01787 ± 0.00466	0.01949 ± 0.00278	0.02077 ± 0.00230	0.01364 ± 0.00218	0.01740 ± 0.00238
**PSVC**	3.9718 ± 0.80786	3.9965 ± 964,870	3.85550 ± 0.52352	3.35619 ± 0.38011	3.59003 ± 0.55426	3.13680 ± 0.8491*

**TABLE 7:** Summary of absolute organ weights for females in the repeat-dose 90-day toxicity study. Values represent mean ± SD. *Statistically significant (*p* < 0.05) with respect to the vehicle group.

Dose (mg/kg b.w.)	Vehicle 0	Paraxanthine - 100	Paraxanthine - 150	Paraxanthine - 185	Caffeine - 150	Caffeine - 185
**Adrenals**	0.07334 ± 0.01640	0.08343 ± 0.01597	0.09413 ± 0.02217*	0.0744 ± 0.01500	0.08975 ± 0.01423	0.09303 ± 0.01822
**Thymus**	0.37544 ± 0.10322	0.32814 ± 0.04639	0.28995 ± 0.07156	0.30999 ± 0.06019	0.27308 ± 0.05827*	0.26947 ± 0.07063*
**Spleen**	0.62254 ± 0.10870	0.60117 ± 0.07442	0.57166 ± 0.08149	0.54832 ± 0.03470	0.56087 ± 0.04883	0.55137 ± 0.09321
**Heart**	1.084 ± 0.34130	1.12372 ± 0.11596	1.16994 ± 0.09185	1.1161 ± 0.06957	1.13286 ± 0.08331	1.14708 ± 0.10927
**Brain**	2.06603 ± 0.08983	2.10052 ± 0.09091	2.00639 ± 0.08111	2.08486 ± 0.07586	1.99639 ± 0.09944	1.94498 ± 0.07350*
**Kidneys**	2.17679 ± 0.23112	2.24008 ± 0.21491	2.10597 ± 0.14674	2.06906 ± 0.23927	2.33549 ± 0.19490	2.09417 ± 0.23091
**Liver**	10.84485 ± 1.58733	10.92884 ± 1.22639	10.68754 ± 1.01925	10.04758 ± 0.76868	10.85476 ± 0.62288	10.60714 ± 0.98894
**Ovaries**	0.15894 ± 0.02590	0.18028 ± 0.04256	0.19807 ± 0.03636	0.13956 ± 0.04787	0.17346 ± 0.04719	0.15929 ± 0.03259
**Uterus with cervix**	1.0486 ± 0.36606	0.72098 0.23428	0.69851 ± 0.17391	0.83874 ± 0.32582	0.77222 ± 0.25966	0.68401 ± 0.28696
**Pituitary**	0.02182 ± 0.00468	0.02260 ± 0.0058	0.02017 ± 0.00248	0.02183 ± 0.00439	0.02064 ± 0.00428	0.01763 ± 0.00515
**Thyroid & Parathyroid**	0.03343 ± 0.00667	0.02438 ± 0.00500	0.02560 ± 0.00677	0.02766 ± 0.00588	0.02599 ± 0.00587	0.03154 ± 0.00987

**TABLE 8:** Summary of absolute organ weights for recovery group males and females in the repeat-dose 90-day toxicity study. Values represent mean ± SD. *Statistically significant (*p* < 0.05) with respect to the vehicle group.

Dose (mg/kg b.w.)	Vehicle 0 recovery (males)	Paraxanthine—185 recovery (males)	Caffeine– 185 recovery (males)	Vehicle 0) recovery (females)	Paraxanthine—185 recovery (females)	Caffeine– 185 recovery (females)
**Adrenals**	0.05552 ± 0.00744	0.07106 ± 0.01002*	0.06319 ± 0.00318	0.06627 ± 0.01083	0.0689 ± 0.01499	0.07309 ± 0.00835
**Thymus**	0.34461 ± 0.09950	0.3429 ± 0.05200	0.31459 ± 0.09923	0.27167 ± 0.10157	0.32416 ± 0.06561	0.29798 ± 0.05752
**Spleen**	0.84257 ± 0.16616	1.02084 ± 0.10001	0.74054 ± 0.16568	0.52665 ± 0.03057	0.55206 ± 0.05684	0.55663 ± 0.09083
**Heart**	1.71727 ± 0.17712	1.98022 ± 0.12241*	1.62546 ± 0.16916	1.06126 ± 0.03013	1.15511 ± 0.14457	1.03703 ± 0.04720
**Brain**	2.22603 ± 0.19378	2.16304 ± 0.09426	2.10495 ± 0.09495	2.06947 ± 0.09111	2.00996 ± 0.09472	1.93851 ± 0.10298
**Kidneys**	3.52317 ± 0.23260	4.05760 ± 0.20928*	3.19812 ± 0.50449	2.05429 ± 0.14511	2.07322 ± 0.27388	1.87379 ± 0.03797*
**Liver**	15.4413 ± 1.82498	16.7718 ± 1.16758	11.68069 ± 1.81996*	8.83301 ± 1.09478	9.87243 ± 1.36150	7.83365 ± 0.70018
**Testes**	3.48985 ± 0.30427	3.8643 ± 0.27753	3.59449 ± 0.33402	n/a	n/a	n/a
**Epididymides**	1.51534 ± 0.16365	1.60369 ± 0.08125	1.44345 ± 0.12122	n/a	n/a	n/a
**PSVC**	3.7983	3.24068*	3.22433	n/a	n/a	n/a
**Thyroid and parathyroid**	0.034 ± 0.00814	0.03344 ± 0.00217	0.03036 ± 0.00493	0.02939 ± 0.00847	0.03114 ± 0.00981	0.02293 ± 0.00443
**Pituitary**	0.02050 ± 0.00425	0.02279 ± 0.00251	0.01523 ± 0.00149*	0.02069 ± 0.00136	0.02933 ± 0.00801*	0.02027 ± 0.00193
**Ovaries**	n/a	n/a	n/a	0.16256 ± 0.02231	0.12835 ± 0.02348*	0.16897 ± 0.00657
**Uterus with cervix**	n/a	n/a	n/a	0.85348 ± 0.33820	0.92171 ± 0.17938	0.69884 ± 0.09278

No external or internal gross pathological changes were observed in any of the animals at all tested dose levels or in the vehicle control groups. In terms of histopathological examination, a single case of decreased cellularity in the sternum of high dose paraxanthine and caffeine-treated males was observed. However, the observation was not considered a direct effect of the test item but secondary to the decreased body weight gain. All other histopathology findings observed in all the study animals were sporadic in nature and lacked consistency (Supplementary Table S9).

## 4 Discussion

**FIGURE 6:**
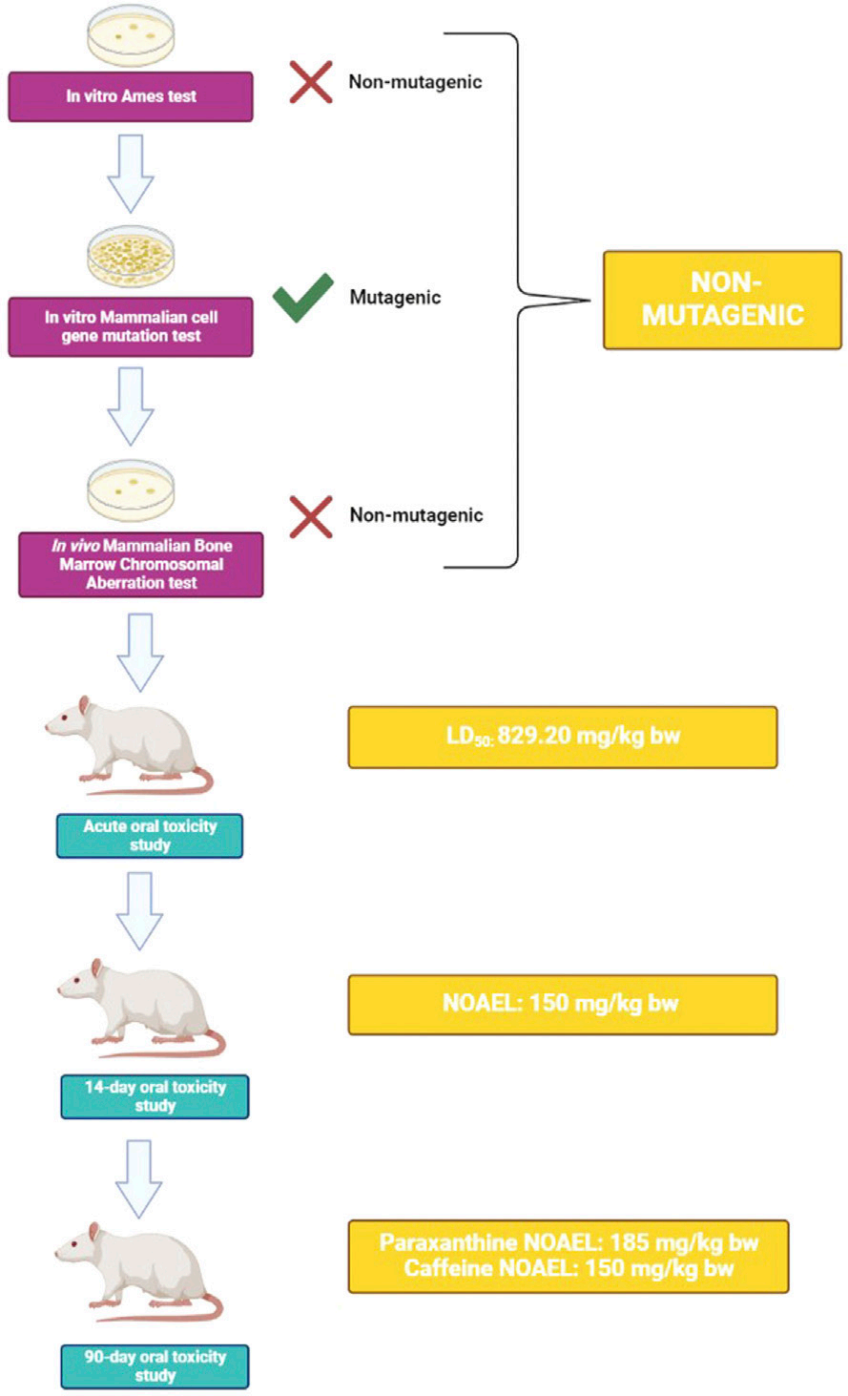
Schematic summary of experimental findings. Results from a battery of genetic toxicity tests demonstrate that paraxanthine is non-mutagenic. In an *in vivo* test, acute toxicity experiments derived an LD_50_ of 829.20 mg/kg bw for paraxanthine. Upon repeated exposure to paraxanthine in a 14-day study, a NOAEL of 150 mg/kg bw was derived. Lastly, in the 90-day subchronic oral toxicity study, the toxicity of paraxanthine and caffeine were compared. Paraxanthine demonstrated a better toxicity profile compared to caffeine, as the established NOAEL was 185 mg/kg bw compared to that of caffeine at 150 mg/kg bw. This figure was created using BioRender.

The battery of toxicological studies conducted on paraxanthine (supplied by Rarebird Inc.) demonstrated a lack of *in vitro* and *in vivo* toxicity. Altogether, results from the three battery genetic toxicology studies (bacterial reverse mutation, mammalian chromosomal aberration, mammalian cell gene mutation) suggest that paraxanthine is non-mutagenic at all doses tested. In the acute oral toxicity study, an LD_50_ of 829.20 mg/kg body weight (bw) was determined for paraxanthine. There was no mortality or treatment-related adverse effects observed for the assessed parameters in the 14-day repeat dose oral toxicity study following daily oral administration of low, mid, or high doses of paraxanthine (50, 100, or 150 mg/kg bw). The same findings were observed in the subchronic repeat-dose 90-day oral toxicity study at daily doses up to 185 mg of paraxanthine/kg bw resulting in the assignment of the highest dose tested as the NOAEL. While there were no reported deaths upon administration of paraxanthine, mortality was reported in two animals in the high dose caffeine-treated group (185 mg/kg bw). Therefore, the NOAEL from the 90-day study was determined to be 150 mg/kg bw for caffeine. These findings are summarized in Figure 6.

The genetic toxicology studies provide evidence that paraxanthine is non-mutagenic. In the Ames test, paraxanthine did not result in an increased number of revertants in five strains of *Salmonella typhimurium* at concentrations up to 3,000 ug/plate. While the *in vitro* mammalian cell gene mutation test suggested that paraxanthine (up to 300 μg/ml) was mutagenic at the TK locus of cultured mouse lymphoma cells in the presence of metabolic activation, the *in vivo* mammalian bone marrow chromosomal aberration test demonstrated a lack of chromosomal aberration and toxicity to the bone marrow of Sprague Dawley rats (doses up to 100 mg/kg bw). When comparing the latter two studies, it is important to recognize the drawbacks of using mammalian cell culture *versus* an *in vivo* model. While cell lines are valuable research tools, the behavior of cell lines can vary in culture in comparison to an intact organism. A similar phenomenon to the findings of these genotoxicity studies has been observed with caffeine, which is mutagenic to mammalian cells in culture but not mutagenic when the mammalian cells are cultured in the presence of liver extracts containing detoxifying enzymes (Nehlig and Debry, 1994). Similarly, coffee and caffeine are devoid of mutagenic effects *in vivo*. Considering that paraxanthine is intended to be an ingredient in human food, the data from the *in vivo* Mammalian Bone Marrow Chromosomal Aberration test better represent the effects of the test item in an intact animal. Therefore, it can be concluded that paraxanthine is non-mutagenic at doses up to 100 mg/kg bw.

While these preclinical studies are important for determining the genetic toxicity potential of paraxanthine, the *in vivo* mammalian bone marrow chromosomal aberration test only involves the administration of a single dose of the test item. Since paraxanthine is intended to be added to foods proposed to be ingested chronically, *in vivo* repeat-dose studies are warranted to better understand the toxicology profile of paraxanthine. In order to establish a dose that can be used for subchronic studies, a maximum-tolerated dose study was first conducted to assess the acute toxicity of paraxanthine. Single dose administration of paraxanthine by oral gavage up to 750 mg/kg/b.w. in female Sprague Dawley rats showed no adverse effects on body weight, or body weight gain. Moreover, gross necropsy examination revealed no treatment related adverse effects. Based on these results, the calculated single dose LD_50_ of paraxanthine was 829.20 mg/kg b. w. Therefore, the LD_50_ of paraxanthine is 2.3-fold higher than the value that has been reported for caffeine (LD_50_ 367 mg/kg b. w.) (Adamson, 2016). Moreover, the LD_50_ of paraxanthine was determined to be 1,601 mg/kg bw in another study in rats, suggesting that the toxicity may be even lower than the levels reported in the present study (Purpura et al., 2021).

Repeat-dose oral toxicity was subsequently assessed in a 14-day study. Rats administered paraxanthine up to 150 mg/kg bw showed no treatment-related adverse effects on body weight, body weight gain, feed consumption, hematology, coagulation or clinical chemistry parameters, organ weights, gross pathological findings, microscopic examination of tissues or clinical signs compared to the vehicle. Although there were significant differences observed in certain parameters (feed consumption, body weight, clinical chemistry parameters), the differences were not toxicologically significant. To better assess the subchronic toxicity of paraxanthine, a repeat-dose 90-day study was subsequently conducted.

In the 90-day repeat dose oral toxicity study, there was no mortality reported in rats that received paraxanthine at doses up to 185 mg/kg bw, while mortality was observed in two rats that received the same dose of caffeine. Although a reduction in body weight gain was observed in paraxanthine-treated rats, reductions in body weight gain have also been noted in other studies where stimulant ingredients have been evaluated, including caffeine (Zheng G. et al., 2004; Greenberg et al., 2006; Tabrizi et al., 2019; Sirotkin and Kolesárová, 2021). In fact, phytochemicals, such as methylxanthines, have been reported to promote body fat reduction and weight loss in obese individuals. The role of methylxanthines on the modulation of adipose tissue function has previously been reported, and methylxanthines have been identified as potential substances for the development of novel therapeutic approaches for obesity treatment (Carrageta et al., 2018). Since the dose-dependent decrease in body weight gain was an expected pharmacological effect of the test item, and recovery of body weight gain was observed during the recovery period, the effect of both test items (caffeine and paraxanthine) on reducing body weight gain was not considered to be an adverse effect (Zheng G. et al., 2004; Kobayashi-Hattori et al., 2005; Sirotkin and Kolesárová, 2021). Moreover, comparatively, the expected/anticipated test item-related pharmacological effects of paraxanthine on body weight and body weight gain were less (less reduction and recovery) compared to caffeine-treated animals. Similar findings were observed in another study where paraxanthine was administered to Wistar rats (Purpura et al., 2021). In this study, the authors also reported reduced feed consumption, body weight and body weight gain. It was suggested that the changes could be due to increased methylxanthine-related changes in thermogenesis and appetite suppression. In addition to methylxanthines contributing to changes in body weight, behavioral changes in paraxanthine-treated animals were also likely mediated by the effects of the methylxanthine. Paraxanthine-treated animals showed clinical signs of hyperactivity and hypereflexia, while caffeine-treated rats demonstrated hyperactivity, burrowing and partial eyelid ptosis. However, the effects were not considered to be adverse events as the clinical signs were completely reversed at the next-day observation. Furthermore, all of these findings have been reported in rodent models exposed to methylxanthines such as caffeine, theophylline and theobromine (Izawa et al., 2010; Dalefield, 2017; Murray and Traylor, 2022).

Some parameters evaluated in clinical chemistry were significantly different in paraxanthine and caffeine-treated animals compared to vehicle controls. However, similar to the changes described above, the differences were considered to be non-toxicologically significant as the changes were either non-dose dependent; the values remained within a normal, healthy physiological range; or differences in values occurred in only one sex. Furthermore, several of these findings represented non-adverse changes because they were accompanied by no histopathological changes or changes in organ weights. The few observed histopathology findings were considered incidental or background to the Sprague Dawley rats used in the study as they were sporadic in nature and lacked consistency (McInnes, 2012). Significant differences in urinalysis parameters of paraxanthine and caffeine-treated rats were considered incidental, and the increase in urine volume might be due to the diuretic effect of the test item (Osswald and Schnermann, 2011).

Overall, it was concluded that were no treatment-related external or internal gross pathological changes, differences in organ weights, or effects on urinalysis, hormonal parameters, hematology, or coagulation in any of the animals at all tested dose levels of paraxanthine relative to the vehicle control groups. These results are in accordance with findings from a previous 90-day toxicity study in Wistar rats, administered paraxanthine from a different ingredient supplier. In this study, the authors reported the NOAEL to be the highest dose (300 mg/kg bw) tested since there were no treatment-related adverse effects (Purpura et al., 2021). Similarly, in the present study, the NOAEL was determined to be the highest dose tested. However, the NOAEL value in the present study was limited by the upper level studied, suggesting that the true subchronic NOAEL may exceed 185 mg/kg, as was demonstrated by Purpura and colleagues (Purpura et al., 2021).

Based on the above findings, the results demonstrate that paraxanthine was safe and well-tolerated under the conditions of these studies, as there were no treatment-related adverse effects of toxicological significance in rats exposed to all doses of paraxanthine. Comparatively, the expected/anticipated test item-related pharmacological effects of paraxanthine on body weight and body weight gain were found to be less (less reduction and recovery) compared to caffeine-treated animals. Moreover, mortality was reported in the caffeine-treated animals while all animals in the paraxanthine group survived the study. In view of the above findings, the NOAEL of paraxanthine was determined to be 185 mg/kg body weight and NOAEL for caffeine was 150 mg/kg body weight in both male and female Sprague Dawley rats following once daily administration for 90 consecutive days by oral gavage under the experimental conditions employed. These findings open future discussions about whether paraxanthine might be safer than caffeine.

## Data Availability

The original contributions presented in the study are included in the article/Supplementary Materials, further inquiries can be directed to the corresponding author.
