# Supplementary material for: Paraxanthine safety and comparison to caffeine
Source: Front Toxicol. 2023 Feb 2;5:1117729. doi: 10.3389/ftox.2023.1117729 (PMC9932512; doi:10.3389/ftox.2023.1117729)
Supplement: Supplementary file 1 [file DataSheet1.docx]

**Table 1. Clinical abnormalities reported in the maximum-tolerated dose study of Rarebird’s paraxanthine.**

| **Dose of paraxanthine (mg/kg b.w.)** | **Clinical signs** |
| --- | --- |
| 100 | There were no adverse clinical signs observed in all the animals during the 15-day observation period. |
| 125 | All animals showed clinical signs of hyper-reflexia up to 2 hours post dose. |
| 200 | Piloerection, hyperactivity, tonic convulsions, salivation, hyperreflexia, hunched posture, hypoactivity, somnolence, decreased response, lacrimation, dehydration and emaciation. |
| 375 | Piloerection, recumbent/prostration, abnormal posture, hypoactivity, somnolence, ataxia, and lacrimation. |
| 500 | Piloerection, hypoactivity, somnolence, abdominal breathing, recumbent/prostration, cyanosis, lacrimation and hyper-reflexia. Mortality was observed in one animal on day 2 post dose. |
| 750 (preliminary) | Abnormal posture, piloerection, hypoactivity, ataxia, lacrimation, somnolence, abdominal breathing, recumbent/prostration and hyper-reflexia. |
| 750 (confirmatory) | Abnormal posture, piloerection, ataxia, hypoactivity, somnolence, abdominal breathing, recumbent/prostration, hyper-reflexia, and lacrimation. |
| 1000 | Hypoactivity, somnolence, abnormal posture, recumbent/prostration, abdominal breathing, decreased response, salivation, piloerection, brownish and transparent discharge from anal opening, and ataxia. One animal was found dead at 2 h post-dose and the remaining two animals were found on day 2. |
| 3750 | Abnormal posture, hypoactivity, somnolence, ataxia, whitish mucus discharge form anal opening, recumbent/prostration, lacrimation, decreased response, cyanosis, tonic-clonic convulsions, abnormal breathing, and gasping. One animal was found dead. The remaining two animals were found to be moribund and were euthanized. |
| 5000 | Hypoactivity, abdominal breathing, cyanosis, recumbent/ prostration, ataxia, tremor, lacrimation, recumbent, nasal discharge, tonic and clonic convulsion, asphyxia convulsion, opisthotonous, gasping. All 3 animals died within 4 hours. |

**Table 2. Summary of clinical chemistry results for males and females in the 14-day repeat dose study.** *Values are expressed as mean ± standard deviation (SD), * Statistically significant at P<0.05 vs. the vehicle control group.*

|  | | **Males** | | | | | **Females** | | | | |
| --- | --- | --- | --- | --- | --- | --- | --- | --- | --- | --- | --- |
| **Dose (mg/kg b.w)** | | **Cholesterol (mg/dL)** | **Ca+ (mg/dL)** | **Phosphorus (mg/dL)** | **K^+^  (mmol/L)** | **ALT (U/L)** | **Cholesterol (mg/dL)** | **Ca+ (mg/dL)** | **Phosphorus (mg/dL)** | **K^+^  (mmol/L)** | **ALT (U/L)** |
| **Vehicle control**  **0.0** | **Mean** | 48.20 | 10.16 | 7.84 | 4.52 | 41.20 | 61.00 | 9.88 | 6.52 | 4.51 | 40.60 |
|  | **SD (±)** | 7.60 | 0.21 | 0.54 | 0.54 | 6.98 | 11.45 | 0.28 | 0.47 | 0.37 | 10.01 |
| **50.0** | **Mean** | 57.60 | 10.10 | 7.73 | 5.08 | 43.60 | 69.00 | 9.42* | 7.00 | 4.07 | 36.00 |
|  | **SD (±)** | 6.23 | 0.19 | 0.34 | 1.57 | 6.69 | 16.79 | 0.33 | 0.44 | 0.26 | 5.10 |
| **100.0** | **Mean** | 63.40* | 9.60* | 7.80 | 4.64 | 53.80* | 68.60 | 9.10* | 6.64 | 4.53 | 45.80 |
|  | **SD (±)** | 5.81 | 0.19 | 0.31 | 0.17 | 3.77 | 3.36 | 0.21 | 0.57 | 0.31 | 10.78 |
| **150.0** | **Mean** | 58.80* | 9.22* | 7.12 | 4.25 | 39.60 | 74.00 | 8.76* | 7.22 | 4.32 | 46.20 |
|  | **SD (±)** | 4.15 | 0.43 | 0.54 | 0.24 | 9.91 | 15.75 | 0.28 | 0.63 | 0.24 | 22.42 |

**Table 3. Summary of functional observation battery test measurements in recovery group males and females in the 90-day repeat dose study.** *The numbers represent the number of animals showing the respective observations. *Statistically significant at P<0.05 vs. the vehicle control group.*

| **Group no.** | | | **Males** | | | **Females** | | |
| --- | --- | --- | --- | --- | --- | --- | --- | --- |
|  |  |  | **Vehicle control** | **Paraxanthine** | **Caffeine** | **Vehicle control** | **Paraxanthine** | **Caffeine** |
| **Dose (mg/kg b.w.)** | | | **0** | **185** | **185** | **0** | **185** | **185** |
| **Number of animals** | | | **5** | **5** | **5** | **5** | **5** | **4** |
| **Open field measurement** | | | | | | | | |
|  | | **Gait** |  |  |  |  |  |  |
| 1 | Normal | | 5 | 5 | 5 | 5 | 5 | 4 |
| 2 | Ataxia, uncoordinated movement, excessive sway, rock, or lurch  (stop and sudden movement) | | 0 | 0 | 0 | 0 | 0 | 0 |
| 3 | Body drags or is flattened (animal’s ventral surface makes contact  with the cart surface) | | 0 | 0 | 0 | 0 | 0 | 0 |
| 4 | Limbs splayed or dragging; unable to support weight (specify hind  or forelimbs) | | 0 | 0 | 0 | 0 | 0 | 0 |
|  | | **Mobility** |  |  |  |  |  |  |
| 1 | Normal (animal moves easily around open field) | | 5 | 5 | 5 | 5 | 5 | 4 |
| 2 | Increased movement (animal moves mostly continuously, rarely  stopping to sniff or groom) | | 0 | 0 | 0 | 0 | 0 | 0 |
| 3 | Decreased movement (reduced movement around field;  movements may be sluggish) | | 0 | 0 | 0 | 0 | 0 | 0 |
| 4 | None (animal does not move around field even after gently  prodding) | | 0 | 0 | 0 | 0 | 0 | 0 |
|  | | **Arousal** |  |  |  |  |  |  |
| 1 | Normal and alert with exploratory movements | | 5 | 5 | 5 | 5 | 5 | 4 |
| 2 | Low; slight stupor, some head or body movements | | 0 | 0 | 0 | 0 | 0 | 0 |
| 3 | Very low; stupor, little or no responsiveness to the environment | | 0 | 0 | 0 | 0 | 0 | 0 |
| 4 | High; slight excitement, tense, sudden darting (sudden rapid  movement) or freezing | | 0 | 0 | 0 | 0 | 0 | 0 |
| 5 | Very high; hyper alert, sudden boost of running or movement | | 0 | 0 | 0 | 0 | 0 | 0 |
|  | | **Stereotype** |  |  |  |  |  |  |
| 1 | None | | 5 | 5 | 5 | 5 | 5 | 4 |
| 2 | Excessive grooming | | 0 | 0 | 0 | 0 | 0 | 0 |
| 3 | Repetitive circling | | 0 | 0 | 0 | 0 | 0 | 0 |
| 4 | Any other | | 0 | 0 | 0 | 0 | 0 | 0 |
|  | | **Piloerection** |  |  |  |  |  |  |
| 1 | Absent | | 5 | 5 | 5 | 5 | 5 | 4 |
| 2 | Present | | 0 | 0 | 0 | 0 | 0 | 0 |
|  | | **Clonic and tonic movement** |  |  |  |  |  |  |
| 1 | Absent | | 5 | 5 | 5 | 5 | 5 | 4 |
| 2 | Present | | 0 | 0 | 0 | 0 | 0 | 0 |
|  | | **Number of Urinations** |  |  |  |  |  |  |
| **Mean** | | | 2.00 | 1.20 | 2.00 | 2.00 | 0.60* | 1.25 |
| **STDEV (±)** | | | 2.00 | 1.30 | 0.71 | 1.58 | 1.34 | 0.96 |
| **Number of Defecations** | | | | | | | | |
| **Mean** | | | 1.20 | 0.60 | 0.40 | 0.00 | 0.00 | 1.00 |
| **STDEV (±)** | | | 1.64 | 0.89 | 0.89 | 0.00 | 0.00 | 0.82 |
| **Number of rearing** | | | | | | | | |
| **Mean** | | | 8.20 | 7.20 | 10.40 | 8.80 | 12.20* | 11.00 |
| **STDEV (±)** | | | 4.76 | 1.64 | 2.61 | 2.17 | 2.05 | 6.38 |

**Table 4. Summary of clinical chemistry results in main group males in the repeat dose 90-day study.** **Statistically significant at P<0.05.*

| **Group & Dose** **(mg/kg b.w.)** |  | **Total protein**  **(g/dL)** | **Globulin**  **(calc)**  **(g/dL)** | **Albumin**  **(g/dL)** | **ALP (U/L)** | **Blood urea nitrogen**  **(mg/dL)** | **Glucose**  **(mg/dL)** | **Total bilirubin**  **(mg/dL)** |  |
| --- | --- | --- | --- | --- | --- | --- | --- | --- | --- |
| **Vehicle control**  **0.0** | **Mean** | 6.90 | 5.75 | 1.15 | 89.60 | 15.10 | 131.90 | 0.10 |  |
|  | **SD (±)** | 0.28 | 0.24 | 0.08 | 24.91 | 1.79 | 7.72 | 0.00 |  |
| **Paraxanthine 100** | **Mean** | 7.10 | 5.88 | 1.22 | 102.30 | 14.80 | 143.90 | 0.13 |  |
|  | **SD (±)** | 0.48 | 0.42 | 0.13 | 17.60 | 2.20 | 16.13 | 0.05 |  |
| **Paraxanthine 150** | **Mean** | 6.74 | 5.63 | 1.11 | 84.70 | 14.60 | 130.30 | 0.15 |  |
|  | **SD (±)** | 0.39 | 0.35 | 0.11 | 13.74 | 2.41 | 19.20 | 0.05 |  |
| **Paraxanthine 185** | **Mean** | 6.92 | 5.77 | 1.15 | 97.10 | 17.30 | 127.70 | 0.13 |  |
|  | **SD (±)** | 0.27 | 0.25 | 0.11 | 21.46 | 3.09 | 17.99 | 0.05 |  |
| **Caffeine 150** | **Mean** | 6.95 | 5.71 | 1.24 | 85.70 | 16.90 | 127.60 | 0.12 |  |
|  | **SD (±)** | 0.56 | 0.46 | 0.16 | 25.06 | 3.67 | 22.83 | 0.06 |  |
| **Caffeine 185** | **Mean** | 7.30 | 6.07 | 1.23 | 113.11 | 16.00 | 120.78 | 0.18 |  |
|  | **SD (±)** | 0.21 | 0.23 | 0.16 | 32.44 | 3.24 | 7.95 | 0.13 |  |

**Table 5. Summary of clinical chemistry results in main group females in the repeat dose 90-day study.****Statistically significant at P<0.05.*

| **Group & Dose (mg/kg b.w.)** | | **Creatine (mg/dL)** | **Albumin**  **(g/dL)** | **Total bilirubin (mg/dL)** | **AST (U/L)** | **ALP (U/L)** | **ALT (U/L)** | **Na^+^ (mmol/L)** | **K^+^ (mmol/L)** | **Cl^-^ (mmol/L)** |
| --- | --- | --- | --- | --- | --- | --- | --- | --- | --- | --- |
|  | **Mean** | 0.31 | 1.48 | 0.09 | 117.90 | 51.70 | 53.50 | 134.90 | 4.18 | 104.33 |
| **Vehicle control**  **0.0** | **SD (±)** | 0.07 | 0.16 | 0.03 | 96.87 | 14.37 | 24.94 | 1.65 | 0.45 | 0.78 |
|  | **Mean** | 0.39 | 1.59 | 0.09 | 137.10 | 56.30 | 55.30 | 135.27 | 4.22 | 104.70 |
| **Paraxanthine 100** | **SD (±)** | 0.10 | 0.38 | 0.06 | 58.42 | 18.76 | 15.47 | 1.13 | 0.22 | 1.79 |
|  | **Mean** | 0.33 | 1.35 | 0.09 | 87.50 | 57.50 | 49.50 | 136.12 | 4.05 | 105.28 |
| **Paraxanthine 150** | **SD (±)** | 0.06 | 0.14 | 0.06 | 11.98 | 17.76 | 10.48 | 1.34 | 0.37 | 0.96 |
|  | **Mean** | 0.30 | 1.37 | 0.10 | 88.10 | 68.50 | 52.60 | 135.51 | 4.17 | 104.81 |
| **Paraxanthine 185** | **SD (±)** | 0.06 | 0.13 | 0.07 | 14.59 | 19.46 | 8.54 | 1.02 | 0.40 | 1.03 |
|  | **Mean** | 0.32 | 1.42 | 0.11 | 92.50 | 65.60 | 64.10 | 135.33 | 4.24 | 104.22 |
| **Caffeine 150** | **SD (±)** | 0.05 | 0.14 | 0.03 | 10.95 | 19.37 | 9.64 | 1.06 | 0.34 | 1.09 |
|  | **Mean** | 0.36 | 1.36 | 0.14 | 92.50 | 74.20 | 62.80 | 134.48 | 4.44 | 103.26 |
| **Caffeine 185** | **SD (±)** | 0.12 | 0.14 | 0.07 | 17.58 | 40.21 | 15.84 | 1.22 | 0.25 | 1.24 |

**Table 6. Summary of urinalysis parameters for main group males and females in the 90-day-repeat dose study**. **Statistically significant at P<0.05.* ***Key:*** *% CC – Percentage coefficient correlation; - Not Applicable; SD (±) - Standard Deviation.*

| **Group No.**  **Dose (mg/kg b.w.)** |  | **Vehicle control**  **0.0** | **Paraxanthine**  **100.0** | **Paraxanthine**  **150.0** | **Paraxanthine**  **185.0** | **Caffeine**  **150.0** | **Caffeine**  **185.0** |
| --- | --- | --- | --- | --- | --- | --- | --- |
|  |  |  | **Males** |  |  |  |  |
| **Volume**  **(mL)** | **Mean** | 12.70 | 12.70 | 25.60* | 28.60* | 20.10 | 11.67 |
|  | **SD (±)** | 2.26 | 1.64 | 15.18 | 12.39 | 11.83 | 5.15 |
|  | **%CC** | - | 0.00 | 101.57 | 125.20 | 58.27 | -8.14 |
| **pH** | **Mean** | 7.60 | 7.30 | 7.25 | 7.25 | 7.40 | 7.39 |
|  | **SD (±)** | 0.70 | 0.26 | 0.26 | 0.26 | 0.61 | 0.22 |
|  | **>=9.0** | - | - | - | - | - | - |
|  | **%CC** | - | -3.95 | -4.61 | -4.61 | -2.63 | -2.78 |
| **Specific gravity** | **Mean** | 1.02 | 1.02 | 1.02 | 1.01 | 1.01 | 1.02 |
|  | **SD (±)** | 0.00 | 0.00 | 0.00 | 0.00 | 0.00 | 0.00 |
|  | **%CC** | - | 0.00 | -0.05 | -0.10 | -0.10 | -0.05 |
|  |  |  | **Females** |  |  |  |  |
| **Volume**  **(mL)** | **Mean** | 13.90 | 11.70 | 8.50 | 8.50 | 16.10 | 10.90 |
|  | **SD (±)** | 4.41 | 1.77 | 2.37 | 3.47 | 5.22 | 5.20 |
|  | **%CC** | - | -15.83 | -38.85 | -38.85 | 15.83 | -21.58 |
| **pH** | **Mean** | 7.25 | 7.11 | 7.10 | 7.25 | 7.25 | 7.60* |
|  | **SD (±)** | 0.26 | 0.21 | 0.21 | 0.49 | 0.26 | 0.39 |
|  | **>=9.0** | - | - | - | - | - | - |
|  | **%CC** | - | -2.07 | -2.07 | 0.00 | 0.00 | 4.83 |
| **Specific gravity** | **Mean** | 1.01 | 1.02* | 1.02* | 1.02 | 1.02 | 1.02* |
|  | **SD (±)** | 0.00 | 0.00 | 0.00 | 0.00 | 0.00 | 0.00 |
|  | **%CC** | - | 0.30 | 0.39 | 0.25 | 0.10 | 0.25 |

**Table 7. Summary of urinalysis parameters for recovery group males and females in the 90-day-repeat dose study.** * *Statistically significant at P<0.05.* ***Key:*** *% CC – Percentage coefficient correlation; - Not Applicable; SD (±) - Standard Deviation.*

|  |  | **Males** | | | **Females** | | |
| --- | --- | --- | --- | --- | --- | --- | --- |
| **Group No.**  **Dose (mg/kg b.w.)** |  | **Vehicle control**  **0.0** | **Paraxanthine**  **185.0** | **Caffeine**  **185.0** | **Vehicle control**  **0.0** | **Paraxanthine**  **185.0** | **Caffeine**  **185.0** |
| **Volume**  **(mL)** | **Mean** | 11.00 | 13.80 | 14.40 | 13.60 | 18.00* | 20.75 |
|  | **SD (±)** | 1.00 | 2.17 | 3.65 | 1.34 | 4.12 | 6.85 |
|  | **%CC** | - | 25.45 | 30.91 | - | 32.35 | 52.57 |
| **pH** | **Mean** | 7.60 | 7.70 | 7.50 | 7.50 | 7.00 | 7.50 |
|  | **SD (±)** | 0.55 | 0.76 | 0.61 | 0.61 | 0.00 | 0.41 |
|  | **>=9.0** | - | - | - | - | - | - |
|  | **%CC** |  | 1.32 | -1.32 |  | -6.67 | 0.00 |
| **Specific gravity** | **Mean** | 1.02 | 1.02 | 1.02 | 1.02 | 1.01 | 1.02 |
|  | **SD (±)** | 0.00 | 0.00 | 0.00 | 0.00 | 0.00 | 0.00 |
|  | **%CC** | - | 0.00 | 0.00 | - | -0.10 | 0.00 |

**Table 8. Summary of clinical chemistry parameters for recovery group females in the 90-day-repeat dose study.** * *Statistically significant at P<0.05.*

| **Dose (mg/kg b.w.)** | **Vehicle (0)** | | **Paraxanthine - 185** | | **Caffeine- 150** | |
| --- | --- | --- | --- | --- | --- | --- |
|  | **Mean** | **SD** | **Mean** | **SD** | **Mean** | **SD** |
| **Total protein (g/dL** | 7.84 | 0.59 | 7.94 | 0.43 | 7.40 | 0.45 |
| **Globulin (calc, g/dL)** | 6.48 | 0.65 | 6.34 | 0.38 | 6.33 | 0.35 |
| **Albumin (g/dL)** | 1.36 | 0.30 | 1.60 | 0.07 | 1.08 | 0.22 |
| **ALP (U/L)** | 36.60 | 14.17 | 50.20 | 31.78 | 37.00 | 3.46 |
| **ALT (U/L)** | 47.60 | 13.24 | 55.20 | 20.84 | 42.25 | 2.87 |
| **AST (U/L)** | 89.60 | 19.27 | 86.40 | 19.93 | 86.00 | 10.42 |
| **Blood urea nitrogen (mg/dL)** | 14.80 | 4.09 | 18.2 | 2.49 | 16.00 | 4.97 |
| **Ca (mg/dL)** | 10.28 | 0.40 | 10.34 | 0.21 | 9.68 | 0.48 |
| **Cholesterol (mg/dL)** | 73.20 | 10.83 | 95.40 | 20.53 | 58.00 | 14.47 |
| **Creatinine (mg/dL)** | 0.44 | 0.03 | 0.50 | 0.05 | 0.47 | 0.04 |
| **HDL (mg/dL)** | 64.60 | 9.76 | 80.40 | 12.18 | 54.00 | 11.43 |
| **LDL (mg/dL)** | 6.60 | 0.89 | 7.20 | 1.92 | 7.50 | 1.73 |
| **GGT (U/L)** | 4.60 | 1.52 | 5.20 | 0.45 | 3.25 | 1.26 |
| **Glucose (mg/dL)** | 128.40 | 9.79 | 127.00 | 4.36 | 119.75 | 4.50 |
| **Phosphorus (mg/dL)** | 4.58 | 0.62 | 4.62 | 0.47 | 5.45 | 0.44 |
| **Total bilirubin (mg/dL)** | 0.16 | 0.05 | 0.16 | 0.05 | 0.18 | 0.05 |
| **Triglycerides (mg/dL)** | 95.80 | 39.98 | 72.60 | 31.35 | 31.75* | 6.13 |
| **Na+ (mmol/L)** | 135.60 | 1.21 | 135.30 | 0.29 | 134.83 | 0.97 |
| **K+ (mmol/L)** | 4.19 | 0.36 | 4.05 | 0.18 | 3.94 | 0.29 |
| **Cl- (mmol/L)** | 105.56 | 1.94 | 106.06 | 0.45 | 105.93 | 0.70 |

**Table 9. Summary of clinical chemistry parameters for recovery group males in the 90-day-repeat dose study.** * *Statistically significant at P<0.05.*

| **Dose (mg/kg b.w.)** | **Vehicle (0)** | | **Paraxanthine - 185** | | **Caffeine- 150** | |
| --- | --- | --- | --- | --- | --- | --- |
|  | **Mean** | **SD** | **Mean** | **SD** | **Mean** | **SD** |
| **Total protein (g/dL** | 7.28 | 0.26 | 6.90 | 0.51 | 7.32 | 0.19 |
| **Globulin (calc, g/dL)** | 6.24 | 0.27 | 5.92 | 0.43 | 6.36 | 0.23 |
| **Albumin (g/dL)** | 1.04 | 0.05 | 0.98 | 0.11 | 0.96* | 0.05 |
| **ALP (U/L)** | 73.2 | 16.39 | 71.40 | 15.96 | 66.8 | 23.21 |
| **ALT (U/L)** | 78.6 | 64.6 | 43.80 | 8.07 | 48.00 | 7.52 |
| **AST (U/L)** | 106.6 | 49.61 | 82.20 | 11.01 | 87.2 | 12.01 |
| **Blood urea nitrogen (mg/dL)** | 16.00 | 2.45 | 17.00 | 0.71 | 14.80 | 1.79 |
| **Ca (mg/dL)** | 10.04 | 0.09 | 9.56 | 0.55 | 9.08* | 0.33 |
| **Cholesterol (mg/dL)** | 51.2 | 3.42 | 59.00 | 15.15 | 54.20 | 10.47 |
| **Creatinine (mg/dL)** | 0.27 | 0.05 | 0.28 | 0.04 | 0.30 | 0.08 |
| **GGT (U/L)** | 4.80 | 1.10 | 4.20 | 1.48 | 5.00 | 1.58 |
| **Glucose (mg/dL)** | 122.20 | 8.87 | 110.60 | 14.06 | 125.80 | 21.48 |
| **Phosphorus (mg/dL)** | 5.52 | 0.16 | 5.98* | 0.30 | 5.56 | 0.22 |
| **Total bilirubin (mg/dL)** | 0.18 | 0.04 | 0.18 | 0.04 | 0.14 | 0.05 |
| **Triglycerides (mg/dL)** | 68.2 | 25.09 | 38.60* | 6.58 | 22.80* | 5.89 |
| **HDL (mg/dL)** | 47.6 | 4.28 | 52.00 | 14.34 | 50.20* | 8.98 |
| **LDL (mg/dL)** | 8.20 | 1.64 | 12.20 | 4.32 | 12.40* | 2.07 |
| **Na+ (mmol/L)** | 142.86 | 0.76 | 140.96 | 1.86 | 140.02* | 1.24 |
| **K+ (mmol/L)** | 4.74 | 0.12 | 4.86 | 0.43 | 4.72 | 0.27 |
| **Cl- (mmol/L)** | 112.04 | 0.68 | 111.16 | 1.35 | 110.00* | 1.05 |

**Table 10. Summary of histopathological examination results.** *Only organs with histopathological findings are presented. Numerical number indicates the number of animals with lesions out of the total number (in parenthesis) of animals; M: Male; F: Female; X: Tissue /organ not evaluated due to sex difference.*

| **Name of Organs / Tissues**  Microscopic observations | **Group and Dose (mg/kg b.w.)** | | | | | | | |
| --- | --- | --- | --- | --- | --- | --- | --- | --- |
|  | **Vehicle control**  **0.0** | | **Paraxanthine**  **185.0** | | **Caffeine**  **185.0** | | | **Caffeine Recovery 185.0** |
| **Sex →**  **No. of animals →** | **M**  **[10]** | **F**  **[10]** | **M**  **[10]** | **F**  **[10]** | **M**  **[10]** | | **F**  **[10]** | **F**  **[05]** |
| **Kidneys** | (10) | (10) | (10) | (10) | (09) | (01) | (10) | (01) |
| Mineralization, unilateral | 0 | 2 | 0 | 1 | 0 | 0 | 0 | 0 |
| **Lung** | (10) | (10) | (10) | (10) | (09) | (01) | (10) | (01) |
| Inflammation, acute bronchioloalveolar | 0 | 0 | 0 | 0 | 1 | 0 | 0 | 0 |
| **Trachea** | (10) | (10) | (10) | (10) | (09) | (01) | (10) | (01) |
| Inflammation, acute | 0 | 0 | 0 | 0 | 1 | 0 | 0 | 0 |
| **Adrenal** | (10) | (10) | (10) | (10) | (09) | (01) | (10) | (01) |
| Vacuolation, cortex, bilateral | 0 | 0 | 1 | 0 | 0 | 0 | 0 | 0 |
| **Prostate & Seminal vesicle with coagulating gland** | (10) | X | (10) | X | (09) | (01) | X | X |
| Prostate- Infiltration, mononuclear cells | 1 | - | 1 | - | 0 | 0 | - | - |
| **Uterus & Cervix** | X | (10) | X | (10) | X | X | (10) | (01) |
| Dilated lumen | - | 3 | - | 2 | - | - | 0 | 0 |
| **Sternum** | (10) | (10) | (10) | (10) | (09) | (01) | (10) | (01) |
| Cellularity, decreased | 0 | 0 | 1 | 0 | 2 | 0 | 0 | 0 |
